# Association Between Neonatal Pain Exposure and Neurodevelopmental Outcomes in Very Preterm Infants During the COVID-19 Pandemic: A Retrospective Observational Study

**DOI:** 10.3390/children13081024

**Published:** 2026-08-01

**Authors:** Yui Shiroshita, Tetsuo Naramura, Mio Kojima, Tetsuya Yoneda, Tomoko Sakaida, Ikuko Sobue

**Affiliations:** 1 Department of Nursing, Faculty of Life Sciences, Kumamoto University, Kumamoto 862-0976, Japan; 2 Division of Neonatology, Perinatal Center, Kumamoto University Hospital, Kumamoto 860-8556, Japan; 3 Department of Nursing Science, Graduate School of Biomedical and Health Sciences, Hiroshima University, Hiroshima 734-8553, Japan; 4 Department of Diagnostic Imaging Technology, Faculty of Life Sciences, Kumamoto University, Kumamoto 862-0976, Japan; 5 Nursing Department, Kumamoto University Hospital, Kumamoto 860-8556, Japan; 6 Department of Nursing, Osaka Dental University, Osaka 573-1121, Japan

**Keywords:** COVID-19, neurodevelopment, pain management, pandemics, premature

## Abstract

**Highlights:**

**What are the main findings?**

- COVID-19-related restrictions on parental visits were not associated with neurodevelopment at corrected 18 months and 36 months.
- Pain exposure during the neonatal period was significantly associated with cognitive development at 36 months.

**What is the implication of the main findings?**

- COVID-19-related parental visitation restrictions may not significantly influence early childhood development, but high exposure to pain during the NICU stay may influence developmental delays.
- Even under pandemic conditions, pain management may remain important for the neurodevelopment of very preterm infants.

**Abstract:**

**Background**: Research on preterm infant development during the coronavirus disease 2019 (COVID-19) pandemic has yielded inconsistent results and has not considered the impact of exposure to pain. This study aimed to investigate the association between neurodevelopment in very preterm infants and pain exposure during the COVID-19 pandemic. **Methods:** This retrospective study included very preterm infants born before 33 weeks’ gestation between 2017 and 2022. Their neurodevelopmental status was assessed using the Kyoto Scale of Psychological Development at corrected 18 months (*n* = 92) and 36 months (*n* = 82) (*n* = 35 and 23 during the COVID-19 pandemic, respectively). **Results:** Multiple regression analysis adjusted for confounding factors showed that the developmental quotient at corrected 18 months was not associated with parental visit frequency or invasive procedure count. At 36 months, it was also not associated with parental visit frequency; however, in the cognitive–adaptive domain, it showed a significant association with the number of invasive procedures (*B* = −0.10; β = −0.45; 95% confidence interval [−0.76, −0.14]; *p* = 0.0049). **Conclusions:** These findings highlight the importance of maintaining neonatal pain management during disease outbreaks to support neurodevelopmental outcomes in very preterm infants.

## 1. Introduction

Preterm birth is associated with long-term neurodevelopmental disorders; the earlier the birth occurs, the higher the risk [1,2]. In preterm infants, the brain continues to develop in an extrauterine environment, where it is exposed to harmful stimuli that may alter the maturation process. Very preterm infants (<33 weeks’ gestation) admitted to the neonatal intensive care unit (NICU) frequently undergo painful procedures for both treatment and diagnostic purposes [3]. Of note, their nociceptive pathways and descending inhibitory control are still immature, making them highly vulnerable to pain [4]. Repeated exposure to pain during this critical period of rapid and complex brain development adversely affects central nervous system (CNS) maturation [5]. A higher number of skin injuries was found to be associated with a reduction in white and gray matter (i.e., impaired myelination) [6]. Additionally, reductions in white and gray matter resulting from pain exposure during infancy may persist into school age [7]. Increased exposure to pain during infancy has also been associated with poorer neurodevelopmental outcomes in cognitive, motor, and behavioral domains, and this association can persist into adolescence and beyond [8,9].

Meanwhile, parent–infant interaction contributes to reducing stress and promoting development in preterm infants. Kangaroo care during NICU hospitalization promotes maturation of the autonomic nervous system [10] and improve neurodevelopment [11] in preterm infants. However, methods without direct parent–infant contact also have positive effects on preterm infant development. For example, the scent of breast milk [12,13] and recordings of the mother’s voice [14] can stabilize physiological functions and promote development in preterm infants. Therefore, parental presence in the NICU may also contribute to the neurodevelopment of these infants [15]. Conversely, separating parents and infants immediately after birth disrupts the parent–child bond [16,17], undermines mother–infant relationships [18,19], and may adversely affect the child’s development. Amid the coronavirus disease 2019 (COVID-19) pandemic, the World Health Organization encouraged uninterrupted skin-to-skin contact between mothers and infants, including preterm infants, as early as after birth while taking preventive measures against infection [20]. Nevertheless, many NICUs worldwide imposed strict visiting restrictions, including banning or limiting parental visits, for infection control [21], thereby reducing parental care for preterm infants admitted to the NICU [22].

Research findings on developmental outcomes in preterm infants during the COVID-19 pandemic are inconsistent. Some studies have reported impairments in cognitive and language development at 18–36 months of corrected age [23,24], while others have noted maintenance in motor, cognitive and language development at 18–24 months of corrected age [25,26]. Still, motor and cognitive development improvements have been observed at 6–12 months of corrected age [27]. These studies, whose findings are inconsistent, have not considered the association with pain exposure. Exposure to pain during the neonatal period has been reported to impair CNS maturation [5] and cause poorer neurodevelopmental outcomes [8,9]. These adverse effects may last longer [8,9] and seriously impact the development of preterm infants. Therefore, when evaluating the impact of visitation restrictions associated with the COVID-19 pandemic on preterm infant development, the effects of pain exposure should also be considered.

This study could be the first to focus on pain exposure in relation to development among preterm infants during the COVID-19 pandemic. This research aimed to clarify the association between pain exposure, as well as parental visitation restrictions at NICU, during the COVID-19 pandemic and development in preterm infants at corrected 18 months and 36 months.

We hypothesized that the frequency of parental visits would be significantly associated with developmental outcomes at corrected 18 months and 36 months, and that the number of invasive procedures during the neonatal period would likewise be significantly associated with developmental outcomes at these ages. In other words, when accounting for the effects of pain exposure, we anticipated that visitation restrictions imposed during the COVID-19 pandemic would negatively influence early childhood development. The findings of this study offer important insights for developing interventions for this vulnerable population experiencing strict NICU restrictions during the COVID-19 pandemic. They also provide a framework for supporting preterm infant development in future infectious disease outbreaks.

## 2. Methods

### 2.1. Study Design

This retrospective study, which adhered to the Strengthening the Reporting of Observational Studies in Epidemiology (STROBE) guidelines for observational research [28], was conducted on very preterm infants born in Kumamoto, Japan. Data were extracted from medical records and analyzed from March 2024 to March 2026.

### 2.2. Participants

**Figure 1:**
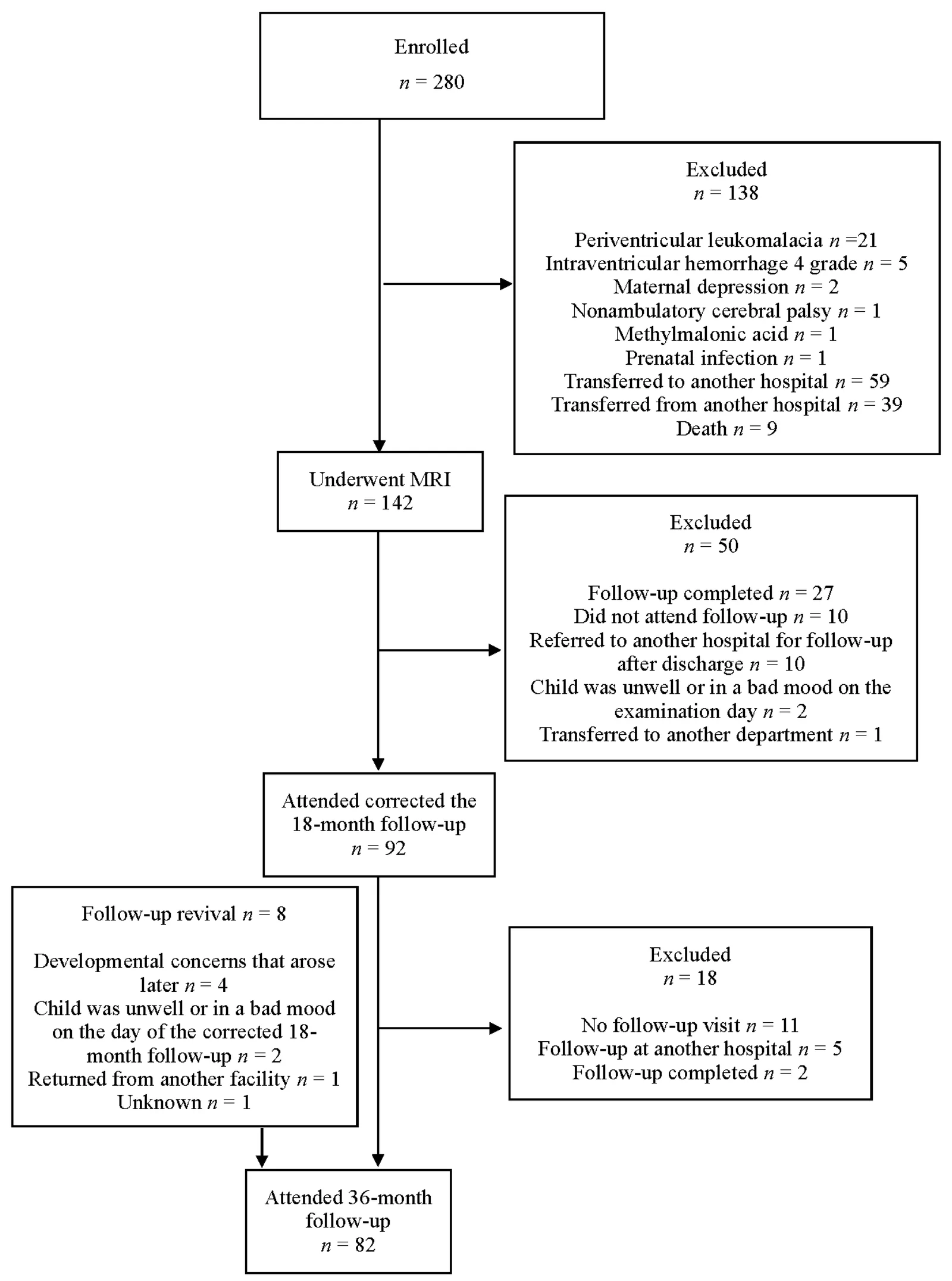
Participant flowchart.

This study included very preterm infants (*N* = 280; gestational age: <33 weeks) born between 2017 and 2022 who were discharged from the Level III NICU at Kumamoto University by 1 May 2023, when COVID-19 was designated as a Category 5 infectious disease in Japan. Of the 280 very preterm infants (Figure 1), 31 were excluded because of the presence of periventricular leukomalacia, grade 3 or 4 intraventricular hemorrhage, maternal depression, nonambulatory cerebral palsy, methylmalonic aciduria, and prenatal infection, because these conditions have been reported to substantially affect neurodevelopmental outcomes [29,30]. We also excluded 59 infants who transferred to other hospitals and 39 who transferred from other hospitals, given the incompleteness in medical record data covering the entire duration of NICU hospitalization. Nine cases of death were also excluded. Out of 142 remaining children, 50 were further excluded because of completion of developmental follow-up; failure to attend the appointment for developmental follow-up; follow-up at other hospitals; failure to undergo assessment because of poor health or a bad mood on the test day; and transfer to another department. A total of 92 children remained for the corrected 18-month developmental assessment and thereby included in the analysis. Of these 92 children, 18 were additionally excluded because of failure to attend the appointment for developmental follow-up, follow-up at other hospitals, and completion of developmental follow-up. Meanwhile, eight infants were re-enrolled for the following reasons: emerging developmental concerns, inability to undergo the modified 18-month follow-up because of poor health or a bad mood on the test day, transfer from another hospital, and unknown reasons. Therefore, 82 children ultimately underwent the 36-month developmental assessment and were thereby included in the analysis.

At the study facility, standard pain management care comprised holding, touching, and facilitated tucking; no oral sucrose was administered. For pharmacological therapy, fentanyl or midazolam was used during scheduled endotracheal intubation; no other medications were used for other procedures. Eutectic mixture of local anesthetics (EMLA) cream and EMLA patches were also not administered.

This study obtained approval from the Kumamoto University Ethics Review Committee (approval number: 2905). Given the study’s retrospective design and utilization of existing clinical data, an opt-out approach was used, with parents provided with an explanation and the opportunity to decline participation. All data were derived from examinations performed as part of routine clinical care, with no procedures conducted specifically for research purposes or beyond standard clinical treatment.

### 2.3. Demographic and Clinical Data Collection and Neurodevelopmental Outcome Assessment

Y.S., T.S. and one NICU nurse independently reviewed the medical records spanning from birth to NICU discharge and extracted the variables.

Pain during the neonatal period was quantified by the number of invasive procedures. Invasive procedures included heel prick, peripheral and central venous punctures, arterial line insertion, subcutaneous injection, intramuscular injection, drain insertion, endotracheal intubation, nasogastric tube insertion, and fundus examinations [5].

The cumulative intravenous (no oral) doses of fentanyl, morphine, and midazolam were calculated by multiplying the average daily dose, adjusted for body weight, by the number of administration days [31].

Additionally, the following data were recorded: gestational age at birth, sex, birth weight, length of NICU stay, Apgar score, number of siblings, mechanical ventilation duration (in days), number of surgical procedures, and surgical history (including laparotomy, hernia repair, colostomy, labial fusion, patent ductus arteriosus closure, and retinal photocoagulation).

Parental visit frequency, defined as the number of visits relative to the length of hospital stay [32], was also calculated. According to previous research, it is the number of parental visits recorded by clinical staff over the total length of each infant’s hospital stay. When the parental visit frequency was close to 1.0, exceeded 1.0, and was below 1.0, the parent visited almost every day, more than once per day (on average), and less than once per day (on average), respectively. Given that the medical records did not contain sufficient details, parental care during visits was limited to the number of kangaroo care sessions. During the COVID-19 pandemic, parental visitation restrictions were implemented from April 2020 to April 2023. Visits were limited to once a day, once every two days, or once a week, depending on the infection situation. Between January and February 2022, parental visits were completely prohibited and permitted only when deemed necessary by a physician. Each visit was limited to a maximum of 15 min. Mothers hospitalized in the maternity ward of the same hospital were allowed a total of three visits per day, each lasting 15 min or less. For predischarge parenting guidance and direct breastfeeding, parental visits were permitted as many times as necessary, lasting up to 60 min per session. Although online visits were introduced in April 2021, none of the parents of the study participants used this option. Before the pandemic, no restrictions on visit duration or frequency were imposed.

Furthermore, data on mothers’ educational attainment, a factor related to child development, were collected [26]. Maternal education has been associated with stimulating parenting practices and early learning opportunities that support child development [33]. This variable was categorized as “high school graduation or less” or “higher than high school graduation” [26].

Preterm infants’ neurodevelopmental outcomes were assessed using the Kyoto Scale of Psychological Development 2001 (KSPD) [34], which is widely used in Japan. Clinical psychologists administered the KSPD during the follow-up examinations of infants at corrected 18 months and 36 months. The developmental quotient (DQ) derived from the overall, postural–motor, cognitive–adaptive, and language–social domains in KSPD was also used for assessing infants’ neurodevelopmental outcomes. A DQ score below 70, equivalent to a Bayley III cognitive score below 85 [35], indicates a significant developmental delay.

### 2.4. Data Analysis

Data were analyzed using R statistical software version 4.3.2. To investigate the effects of COVID-19-associated parental visitation restrictions and pain exposure during NICU admission on developmental outcomes in early childhood, we conducted multiple regression analysis. The primary exposure variables were the number of invasive procedures during NICU admission and the frequency of parental visits. On the basis of prior research and clinical findings, we preselected candidate confounding factors associated with both the primary exposure variables and early childhood developmental outcomes. The correlations among the variables were then evaluated using Spearman’s correlation analysis. Variables with a correlation coefficient exceeding 0.8 were evaluated for inclusion in the analytical model while considering potential multicollinearity. Mechanical ventilation duration (in days) strongly correlated with the invasive procedure count (*r* = 0.8163). In this study, the invasive procedures included endotracheal intubation for mechanical ventilation; therefore, the mechanical ventilation duration was considered to be closely related to the number of invasive procedures. The length of hospital stay also showed a strong correlation (*r* = 0.9314) with the number of invasive procedures. Consequently, we retained the number of invasive procedures—the primary exposure variable—in the final analysis model and excluded the number of days on mechanical ventilation and the length of NICU stay. Furthermore, birth weight strongly correlated with gestational age (*r* = 0.8027). In the present study, we used gestational age only because it is a more direct indicator of the infant’s maturity, in line with previous studies [36,37]. In addition, only eight infants received morphine, and all of whom were also receiving fentanyl. Thus, the pharmacokinetic exposure variables used were the cumulative doses of fentanyl and midazolam. Of note, morphine equivalents calculated from a mixture of fentanyl and morphine were not used because this method has not been established for newborns [38]. For the multiple regression analysis, the final independent variables included gestational age at birth [24,25,36,37,39,40,41], sex [40], surgery history during NICU admission [42,43], fentanyl exposure [42,43], midazolam exposure [42], age during the examination [25], and maternal education [26,37,39].

All variance inflation factors (VIFs) were within the acceptable range (VIF < 5). Missing data were handled using complete-case analysis, and preterm infants with missing values for any of the variables were excluded from each analysis. To interpret the associations’ magnitude, we calculated the unstandardized regression coefficients (*B*) and 95% confidence intervals. Standardized regression coefficients (β) were also calculated to indicate the relative strength of the associations among the explanatory variables. Linearity, homoscedasticity, and normality of residuals showed no significant violations according to the Residuals vs. Fitted plot, Scale–Location plot, Q-Q plot, and the Breusch–Pagan test. The points of influence were evaluated using Cook’s distance, and cases with values exceeding 4/n were identified. Given that the multiple regression analyses were conducted at two time points across four developmental domains, we applied Bonferroni correction, with a *p*-value < 0.00625 (0.05/8) considered statistically significant.

For sensitivity analyses, we conducted multiple regression analysis by replacing the independent variable “parental visit frequency” with “COVID-19 pandemic status,” which influenced the frequency of family visits, and “kangaroo care frequency,” another indicator of parent–child contact, to verify the analysis results’ robustness. Kangaroo care was assessed using the frequency of kangaroo care [32]. To account for the infant’s pain severity and treatment effects, we conducted sensitivity analyses for the primary analyses of corrected 18 months and 36 months development by adding the following as covariates: mechanical ventilation duration (in days), NICU stay duration (in days), 5 min Apgar score, morphine dose administered, and birth weight. We also conducted multiple regression analyses focusing on preterm infants weighing below 1500 g at birth to verify whether the results were consistent even in this high-severity subgroup. Moreover, a multivariate analysis incorporating the corrected 18-month developmental index into the primary analysis of 36-month development was conducted to evaluate the associations when considering development at corrected 18 months. According to an assessment of influence points, we conducted an analysis excluding cases with a Cook’s distance exceeding 4/n and examined the differences in results between this analysis and the primary analysis.

## 3. Results

Of the 142 very preterm infants included in the study, 92 (35 during the COVID-19 pandemic) and 82 (23 during the COVID-19 pandemic) underwent developmental assessments at corrected 18 months and 36 months, respectively (Table 1).

Regarding background factors, the excluded infants (*n* = 50) had significantly higher gestational age at birth, birth weight, and 1- and 5 min Apgar scores than the study participants (*n* = 92). Conversely, NICU stay duration, invasive procedure count during the NICU stay, surgery count during NICU admission, surgery history during NICU admission, endotracheal intubation duration, endotracheal intubation history, fentanyl exposure, midazolam exposure, morphine exposure, parental visit count, parental visit frequency, and kangaroo care sessions were significantly lower in the excluded infants than in the study participants (Table 2).

### 3.1. Results on the Association Between DQ and Parental Visit Frequency and Pain Exposure

Adjusted multiple regression analysis showed no significant associations between DQ at corrected 18 months and parental visit frequency or the number of invasive procedures in any domain (Table 3). However, the regression coefficient for the invasive procedures was negative in the cognitive–adaptive domain.

Likewise, the 36-month DQ score was not significantly associated with parental visit frequency in all domains. However, it was significantly associated with the number of invasive procedures in the cognitive–adaptive domain (*B* = −0.10; β = −0.45; 95% CI [−0.76, −0.14]; *p* = 0.0049) (Table 4).

### 3.2. Sensitivity Analysis Results

The sensitivity analysis results are presented in the Supplementary Tables S1–S17. In preterm infants with a birth weight below 1500 g (*n* = 84 for corrected 18 months, *n* = 75 for 36 months), DQ in the cognitive–adaptive domain at 36 months was significantly associated with the number of invasive procedures (*B* = −0.12, β = −0.52, *p* = 0.0021), consistent with the results of the primary analysis (Tables S1 and S2).

The results of analyses wherein mechanical ventilation duration, NICU stay duration, and birth weight were each added to the primary analysis showed no significant association between DQ and either parental visit frequency or the invasive procedure count (Tables S3–S8). The results of adding the 5 min Apgar score to the primary analysis revealed a significant association between DQ in the cognitive–adaptive domain at 36 months and the invasive procedure count (*B* = −0.10, β = −0.46, *p* = 0.0058), in line with the primary analysis results (Tables S9 and S10).

In the sensitivity analysis wherein the categories of parental visit frequency and whether the period was before or during the COVID-19 pandemic were swapped, DQ in the cognitive–adaptive domain was significantly associated with the invasive procedure count at 36 months (*B* = −0.10, β = −0.46, *p* = 0.0044), consistent with the primary analysis results (Tables S11 and S12). Additionally, in another sensitivity analysis wherein the parental visit frequency was swapped with the kangaroo care frequency, DQ in the cognitive–adaptive domain at 36 months was also significantly associated with the invasive procedure count (*B* = −0.11, β = −0.49, *p* = 0.0019) (Tables S13 and S14).

Moreover, the multivariate analysis, which added the corrected 18-month developmental index to the primary analysis of development at 36 months, showed a significant association between the corrected 18-month developmental index and the 36-month DQ; however, neither the parental visit frequency nor the invasive procedure count was significantly associated with the 36-month DQ (Table S15).

According to Cook’s distance evaluation, 5 cases at the 18-month follow-up and 8 cases at the 36-month follow-up exceeded the 4/n threshold, thereby judged as influence points. In a sensitivity analysis excluding these cases, the invasive procedure count remained significantly associated with the DQ in the “overall” domain (*B* = −0.10, β = −0.48, *p* = 0.0033) and the cognitive–adaptation domain (*B* = −0.11, β = −0.51, *p* = 0.0019) at 36 months (Tables S16 and S17).

## 4. Discussion

This study revealed that COVID-19-associated parental visitation restrictions did not significantly affect early childhood development, but high exposure to pain during the NICU stay was associated with poorer developmental outcomes. The findings of this study partly diverged from the initial hypothesis. To our knowledge, this research is the first to examine the relationship between pain exposure and developmental outcomes in preterm infants admitted to the NICU during the COVID-19 pandemic.

### 4.1. Association Between Neonatal Pain Exposure and Delayed Development in Early Childhood

We examined the association between the development of very preterm infants and the effects of pain exposure during the COVID-19 pandemic. The DQ at corrected 18 months and 36 months was not associated with parental visit frequency. In contrast, the number of invasive procedures showed a negative association with the DQ in the cognitive–adaptive domain at corrected 18 months (β = −0.37, *p* = 0.0336), although this association did not remain statistically significant after adjustment for multiple comparisons. At 36 months, the number of invasive procedures was significantly associated with a lower DQ in the cognitive–adaptive domain (β = −0.45, *p* = 0.0049). In preterm infants, pain pathways are underdeveloped [4], and repeated exposure to pain during a period of complex and rapid brain development adversely affects CNS development [8]. Similarly, repeated pain stimuli in the NICU affect the developing brain [8]. Repeated pain exposure has been associated with changes in white matter microstructure and metabolic abnormalities in the subcortical gray matter [6]. In addition, changes in white matter microstructure have been linked to subsequent impairments in executive function and cognitive development [44]. The association between pain exposure in infancy and poorer cognitive developmental outcomes in early childhood observed in this study is consistent with previous research [5,8].

In this research, we used the number of invasive procedures as an indicator of pain exposure experienced during the NICU stay and examined its association with developmental outcomes. In the primary analysis, the invasive procedure count was associated with the DQ score in the cognitive–adaptive domain at 36 months; however, this association disappeared in models that were further adjusted for the mechanical ventilation duration and NICU stay duration individually. However, the mechanical ventilation duration and NICU stay duration strongly correlated with the invasive procedure count; when these variables were included in the model, the VIF exceeded 5, suggesting the presence of multicollinearity. Consequently, in the analysis adjusted for the mechanical ventilation duration and NICU stay duration, the independent association with the number of invasive procedures may have been underestimated. Meanwhile, the association between pain exposure during NICU admission and developmental delays in the cognitive–adaptive domain at 36 months was similarly observed in the sensitivity analyses of preterm infants with a birth weight below 1500 g—who were likely to be in severe condition—among the study participants, as well as in the sensitivity analyses incorporating the 5 min Apgar score in the primary analysis. Thus, the association between the invasive procedure count and developmental outcomes may reflect not only the effects of invasive procedures themselves but also the possibility that severity contributed to the observed association. Therefore, the potential influence of severity should also be considered. Nonetheless, because the invasive procedure count is considered to reflect cumulative exposure to invasive procedures during the NICU stay, it may have the potential to serve as an important clinical indicator for assessing the developmental prognosis of preterm infants.

Furthermore, the association between the invasive procedure count and DQ was indicated by an unstandardized regression coefficient (*B*) of −0.10, suggesting that, after adjusting for other covariates, the DQ decreases by 0.10 points on average for each additional invasive procedure. Preterm infants receive approximately 14 invasive procedures per day during their NICU stay [8] and roughly 643 invasive procedures during the first 4 weeks of their NICU stay [45]. in the present study, the infants received a total of 112.75 ± 66.25 invasive procedures (range: 32–331) throughout their NICU stay. Preterm infants, whose nervous system is still immature, receive more invasive procedures for medical treatment than term infants; consequently, they are at a higher risk for neurodevelopmental abnormalities [46,47]. Therefore, cumulative exposure to pain has a significant impact on developmental delays. Moreover, the number of invasive procedures received by a preterm infant is a factor that can be modified in clinical practice. A study examining the impact of differences in invasive procedure count on development reported that reducing the number of blood draws through the use of an arterial line during NICU admission resulted in higher cognitive scores in early childhood compared with repeatedly inserting a needle for blood extraction [37]. Consequently, the association found in this study between the invasive procedure count during NICU hospitalization and developmental delays in early childhood underscores the importance of minimizing invasive procedures in the NICU as much as possible.

### 4.2. Relationship Between Parental Visitation Restrictions During the Pandemic and Child Development

Reports on preterm infant development during the COVID-19 pandemic are limited, with inconsistent findings [23,24,25,26,27]. Additionally, their analyses did not consider the effects of pain exposure on the development of this age group. After examining the effects of COVID-19–related parental visitation restrictions on child development while considering the impact of pain exposure, we found that the frequency of parental visits was not associated with DQ scores at the corrected 18-month and 36-month time points. Therefore, parental visitation restrictions may not have significantly affected the early childhood development of preterm infants.

We used “parental visit frequency” as an indicator of visitation restrictions, in line with previous research [32]. However, while parental visit frequency reflects the quantity of parent–infant contact opportunities, it is not a direct indicator of parental visit duration or parent–infant interaction quality. The effects of parent–infant contact on the development of preterm infants are not limited to direct contact, such as kangaroo care. Indirect contact, such as the scent of breast milk [12,13] and the mother’s voice [14], has also been shown to stabilize physiological functions and promote development in preterm infants. Therefore, we used parental visit frequency because it reflects opportunities for both direct and indirect contact between parents and infants. Additionally, at the facility studied, parental visit duration during the COVID-19 pandemic was limited to 15 min or less per visit, whereas the pre-pandemic duration was unrestricted. Additionally, a sensitivity analysis wherein “parental visit frequency” was replaced with “before or during the COVID-19 pandemic” yielded results largely consistent with those of the primary analysis. Based on these results, “parental visit frequency” may be considered an indicator reflecting not only parental visitation restrictions associated with the COVID-19 pandemic but also opportunities for direct and indirect parent–child contact. These findings suggest that parental visitation restrictions associated with the COVID-19 pandemic may not have had a significant impact on the early childhood development of preterm infants.

Pain exposure during the neonatal period may have a detrimental effect on a child’s development regardless of the benefits of parent–child physical contact. A multicenter study compared the internalizing and externalizing problem behaviors at corrected 18 months according to the levels of “infant pain management care” (e.g., pain intervention) and “infant-centered care” (e.g., kangaroo care). Results showed that preterm infants in facilities with a high level of “infant pain management care” had lower internalizing problem scores than those in facilities with a low level [48]. In terms of the level of “infant-centered care,” the internalizing and externalizing problem scores did not differ [48]. These findings support the importance of minimizing painful procedures and optimizing pain management.

Development is influenced by various factors, including not only medical factors and pain exposure during NICU hospitalization but also the caregiving environment after discharge. The effects of early-life experiences on developmental outcomes may also differ depending on the age at which development is assessed. While kangaroo care improves cognitive and motor development between 6 months and corrected 12 months of age [49,50], no improvement in cognitive, motor, or social development has been reported at 24–36 months of age [51,52]. The effect of the COVID-19 pandemic on development in healthy young children can persist at 3 years or older, resulting in developmental delays by age 5 [53]. In the present study, developmental assessments were conducted at corrected ages of 18 months and 36 months. One possible explanation for our findings is that although parental visitation restrictions during the COVID-19 pandemic reduced opportunities for parent–infant contact in the NICU, parent–child interaction after discharge may have increased because of social distancing measures, thereby mitigating potential adverse effects on development. To account for the influence of the post-discharge home environment, maternal educational attainment was included as a covariate in the analysis. After adjustment, the frequency of painful procedures remained significantly associated with developmental outcomes.

These findings suggest that although parental visitation restrictions during the COVID-19 pandemic reduced opportunities for parent–child contact in the NICU, they may not have had a substantial impact on early childhood development. Furthermore, our findings suggest that pain management in the NICU, including reducing the number of skin punctures, may remain important even during infectious disease pandemics.

### 4.3. Limitations

This study has several limitations. First, the sample size was small, and significant differences in baseline characteristics were observed between the study participants and those who were excluded. The excluded group included many infants with milder clinical characteristics, including higher gestational age and birth weight and lower exposure to invasive procedures and intensive care. These infants had completed developmental follow-up, whereas the study participants generally required prolonged NICU hospitalization and long-term follow-up. Consequently, the study participants were exposed to parental visitation restrictions for a longer period, and this study evaluated a population with relatively prolonged exposure to NICU visitation restrictions. Therefore, selection bias cannot be ruled out, and caution is warranted when generalizing these findings to all preterm infants admitted to the NICU.

Second, some variables contained missing data. Although we conducted the analysis using the available data, the possibility of bias resulting from the missing data cannot be ruled out.

Third, this study was retrospective and observational in design; although we adjusted for several potential confounders, the influence of residual confounding cannot be ruled out. Therefore, a causal relationship between the number of invasive procedures and developmental outcomes cannot be established. Furthermore, while the number of invasive procedures is an indicator of cumulative exposure to such procedures during NICU hospitalization, it is also closely associated with severity. Although infants with severe IVH and PVL were excluded from the study, residual confounding due to unmeasured prematurity-related clinical factors cannot be completely ruled out. Consequently, completely separating the effects of the number of invasive procedures from those attributable to severity was difficult.

Fourth, in line with previous research [32], this study used parental visit frequency as an indicator of parental visitation restrictions associated with the COVID-19 pandemic. As a result, we could not examine the potential impact of parent–child contact quality and duration—factors that cannot be fully captured by parental visit frequency alone—on developmental outcomes.

Nevertheless, to our knowledge, this study is the first to include the effects of pain exposure in the assessment of preterm infant development following the COVID-19 pandemic, thereby providing important insights. Notably, our findings demonstrate the importance of pain management during NICU admission and do not imply acceptance of parental visit restriction during an infectious disease pandemic. In the future, studies using larger samples, multicenter collaborative studies, prospective studies that distinguish more precisely between severity and exposure to invasive procedures, and long-term follow-up studies to examine the effects of COVID-19-related NICU visitation restrictions on development beyond early childhood are warranted.

## 5. Conclusions

Visitation restrictions during the COVID-19 pandemic were not significantly associated with development among preterm infants. However, high exposure to pain during infancy was associated with poorer developmental outcomes in early childhood. Therefore, these findings highlight the importance of minimizing exposure in the NICU, even during a pandemic.

## Figures and Tables

**Table 1:** Demographic data of participants (*n* = 92).

	Mean	SD	Minimum	Maximum	*N*
Group: During the COVID-19 pandemic, *n* (%)	35	38.04	–	–	92
Gestational age at birth (weeks)	27.98	2.81	23	32	92
Sex: Female, *n* (%)	42	45.65	–	–	92
Birth weight (g)	1058.68	418.98	360	2556	92
NICU stay duration (days)	109.74	45.09	31	263	92
Apgar score at 1 min (points)	3.90	2.13	1	8	90 ^a^
Apgar score at 5 min (points)	6.10	1.81	1	9	90 ^a^
Sibling count (persons)	0.98	0.91	0	3	90 ^a^
Invasive procedure count during NICU stay (times)	112.75	66.25	32	331	92
Surgery count during NICU stay (times)	0.74	1.21	0	6	92
Surgery history during NICU stay, *n* (%)	35	38.04	–	–	92
Duration of mechanical ventilation (days)	21.22	23.49	0	92	92
Intubation history, *n* (%)	83	90.22	–	–	92
Fentanyl exposure (mg/kg)	0.17	0.22	0	1.56	92
Midazolam exposure (mg/kg)	4.04	17.65	0	126.33	92
Morphine exposure (mg/kg)	0.17	0.64	0	3.48	92
Parental visit count (visits)	57.01	47.38	10	238	92
Parental visit frequency (visits/day)	0.50	0.28	0.08	1.18	92
Maternal education: higher than high school graduation, *n* (%)	48	52.17	–	–	74
Kangaroo care (times)	0.52	0.78	0	3	92
Corrected 18-month KSPD 2001	–	–	–	–	–
Corrected age at examination (months)	18.75	1.53	15	25	92
DQ for all domains (points)	89.12	15.16	22	115	92
DQ for the Posture–Motor domain (points)	87.88	19.84	34	154	91 ^a^
DQ for the Cognitive–Adaptive domain (points)	90.18	15.66	25	117	92
DQ for the Language–Social domain (points)	89.49	16.45	31	131	92
36-Month KSPD 2001, *n* (%)	82	89.13	–	–	–
Age at examination (months)	37.87	2.69	35	49	82
DQ for all domains (points)	81.17	14.91	31	108	82
DQ for the Posture–Motor domain (points)	85.19	22.34	27	125	81 ^b^
DQ for the Cognitive–Adaptive domain (points)	81.22	15.34	32	116	82
DQ for the Language–Social domain (points)	81.02	16.95	29	113	82

Invasive procedures during NICU hospitalization include heel pricks, peripheral and central venous catheterization, arterial line insertion, subcutaneous injections, intramuscular injections, drain insertion, endotracheal intubation, nasogastric tube insertion, and fundus examinations. COVID-19, coronavirus disease 2019; DQ, developmental quotient; KSPD, Kyoto Scale of Psychological Development; NICU, neonatal intensive care unit; SD, standard deviation. –, Not applicable. ^a^ The total subsample was less than 92 because some data were missing from the medical records or could not be recorded because of the child’s condition. ^b^ The total subsample was less than 82 because some data were missing from the medical records or could not be recorded because of the child’s condition.

**Table 2:** Comparison of demographic data between study participants and excluded infants.

	Study Participants (*n* = 92)				Excluded Infants (*n* = 50)				*p* Value *
	Mean	SD	Min	Max	Mean	SD	Min	Max	
Group: During the COVID-19 pandemic, *n* (%)	35	38.04	–	–	35	70	–	–	1.0000 ^$^
Gestational age at birth (weeks)	27.98	2.81	23	32	30.41	1.95	24	32	<0.0001
Sex: Female, *n* (%)	42	45.65	–	–	20	40	–	–	0.0052 ^$^
Birth weight (g)	1058.68	418.98	360	2556	1437.43	370.16	584	2068	<0.0001
NICU stay duration (days)	109.74	45.09	31	263	80.18	45.16	34	276	<0.0001
Apgar score at 1 min (points)	3.90	2.13	1	8	5.19	2.11	1	9	0.0010
Apgar score at 5 min (points)	6.10	1.81	1	9	6.81	1.78	2	10	0.0275
Sibling count (persons)	0.98	0.91	0	3	1.10	0.96	0	4	0.4925
Invasive procedure count during NICU stay (times)	112.75	66.25	32	331	67.10	46.60	6	233	<0.0001
Surgery count during NICU admission (times)	0.74	1.21	0	6	0.29	0.96	0	6	0.0037
Surgery history during NICU admission, *n* (%)	35	38.04	–	–	7	14	–	–	<0.0001 ^$^
Duration of mechanical ventilation (days)	21.22	23.49	0	92	7.41	13.74	0	59	<0.0001
Intubation history, *n* (%)	83	90.22	–	–	31	62	–	–	<0.0001 ^$^
Fentanyl exposure (mg/kg)	0.17	0.22	0	1.56	0.09	0.28	0	1.95	<0.0001
Midazolam exposure (mg/kg)	4.04	17.65	0	126.33	0.87	3.03	0	19.02	0.0218
Morphine exposure (mg/kg)	0.17	0.64	0	3.48	0	0	0	0	0.0358
Parental visit count (visits)	0.57	0.47	0.01	2.38	0.32	0.35	0.02	2.13	<0.0001
Parental visit frequency (visits/day)	0.50	0.28	0.08	1.18	0.39	0.23	0.05	0.97	0.0111
Maternal education: higher than high school graduation, *n* (%)	48	52.17	–	–	NA	NA	–	–	–
Kangaroo care (times)	0.52	0.78	0	3	0.14	0.46	0	2	0.0010

* Wilcoxon rank-sum test, ^$^ Chi-square test. Invasive procedures during NICU hospitalization include heel pricks, peripheral and central venous catheterization, arterial line insertion, subcutaneous injections, intramuscular injections, drain insertion, endotracheal intubation, nasogastric tube insertion, and fundus examinations. COVID-19, coronavirus disease 2019; NICU, neonatal intensive care unit; SD, standard deviation. –, not applicable; NA, not available. Maternal educational attainment was assessed by parental questionnaire at the corrected 18-month follow-up visit. Therefore, these data were unavailable for the excluded participants who did not undergo the corrected 18-month follow-up assessment.

**Table 3:** Results of multiple regression analysis on the developmental quotient in the corrected 18-month Kyoto Scale of Psychological Development.

	*B*	β	*p* Value	95% CI	
Overall (*n* = 92)					
Gestational age	−0.73	−0.14	0.3864	−0.45	0.18
Sex	−2.87	−0.09	0.3764	−0.31	0.12
Invasive procedure count	−0.09	−0.38	0.0246	−0.72	−0.05
Surgical history	−3.76	−0.12	0.4485	−0.44	0.20
Fentanyl exposure	−0.20	−0.003	0.9821	−0.27	0.26
Midazolam exposure	0.15	0.17	0.2913	−0.15	0.50
Parental visit frequency	−0.02	−0.03	0.7973	−0.27	0.21
Age during the examination	0.15	0.01	0.9000	−0.22	0.25
Maternal education	−2.36	−0.07	0.4871	−0.29	0.14
*R*^2^					0.2400
Adjusted *R*^2^					0.1331
Postural–Motor ( *n *= 91)					
Gestational age	−1.07	−0.15	0.3702	−0.49	0.18
Sex	−5.91	−0.15	0.1958	−0.38	0.08
Invasive procedure count	−0.11	−0.35	0.0525	−0.71	0.00
Surgical history	−7.36	−0.18	0.2917	−0.52	0.16
Fentanyl exposure	−5.10	−0.06	0.6869	−0.34	0.23
Midazolam exposure	0.03	0.03	0.8592	−0.32	0.38
Parental visit frequency	0.04	0.06	0.6707	−0.20	0.32
Age during the examination	3.36	0.26	0.0413	0.01	0.51
Maternal education	1.30	0.03	0.7844	−0.20	0.26
*R*^2^					0.2650
Adjusted *R*^2^					0.1617
Cognitive–Adaptive ( *n *= 92)					
Gestational age	−0.79	−0.14	0.3839	−0.46	0.18
Sex	−1.92	−0.06	0.5780	−0.28	0.16
Invasive procedure count	−0.09	−0.37	0.0336	−0.72	−0.03
Surgical history	−3.60	−0.11	0.4954	−0.44	0.21
Fentanyl exposure	4.05	0.06	0.6731	−0.21	0.33
Midazolam exposure	0.13	0.15	0.3864	−0.19	0.48
Parental visit frequency	−0.03	−0.05	0.6914	−0.30	0.20
Age during the examination	0.25	0.02	0.8414	−0.22	0.26
Maternal education	−3.60	−0.11	0.3199	−0.33	0.11
*R*^2^					0.2119
Adjusted *R*^2^					0.1011
Language–Social ( *n *= 92)					
Gestational age	−0.05	−0.01	0.9570	−0.33	0.31
Sex	−3.79	−0.12	0.2970	−0.33	0.10
Invasive procedure count	−0.08	−0.33	0.0564	−0.68	0.01
Surgical history	1.81	0.05	0.7440	−0.27	0.38
Fentanyl exposure	−6.95	−0.09	0.4914	−0.37	0.18
Midazolam exposure	0.19	0.20	0.2357	−0.13	0.54
Parental visit frequency	0.00	0.00	0.9879	−0.25	0.25
Age during the examination	−0.61	−0.06	0.6373	−0.30	0.18
Maternal education	−0.69	−0.02	0.8549	−0.24	0.20
*R*^2^					0.1831
Adjusted *R*^2^					0.0682

CI, confidence interval; Bonferroni-corrected significance threshold: *p* < 0.00625. *B*, unstandardized regression coefficient; β, standardized regression coefficient. Only maternal education had missing values (*n* = 18).

**Table 4:** Results of multiple regression analysis on the developmental quotient in the 36-month Kyoto Scale of Psychological Development.

	*B*	β	*p* Value	95% CI	
Overall (*n* = 82)					
Gestational age	0.19	0.03	0.8091	−0.25	0.32
Sex	−9.21	−0.31	0.0021 *	−0.50	−0.12
Invasive procedure count	−0.09	−0.40	0.0180	−0.73	−0.07
Surgical history	−5.36	−0.18	0.2512	−0.48	0.13
Fentanyl exposure	4.08	0.08	0.4778	−0.14	0.30
Midazolam exposure	0.01	0.01	0.9143	−0.20	0.22
Parental visit frequency	−0.02	−0.04	0.7217	−0.25	0.18
Age during the examination	−0.44	−0.08	0.4619	−0.29	0.13
Maternal education	3.19	0.10	0.2800	−0.09	0.29
*R*^2^					0.4116
Adjusted *R*^2^					0.3348
Postural–Motor ( *n *= 81)					
Gestational age	−0.92	−0.11	0.4450	−0.41	0.18
Sex	−8.11	−0.18	0.0765	−0.38	0.02
Invasive procedure count	−0.10	−0.30	0.0845	−0.64	0.04
Surgical history	−13.55	−0.30	0.0641	−0.61	0.02
Fentanyl exposure	−2.44	−0.03	0.7833	−0.26	0.20
Midazolam exposure	0.07	0.04	0.7252	−0.18	0.26
Parental visit frequency	0.00	0.01	0.9612	−0.22	0.23
Age during the examination	0.77	0.09	0.4000	−0.13	0.31
Maternal education	−0.93	−0.02	0.8383	−0.22	0.18
*R*^2^					0.3189
Adjusted *R*^2^					0.2288
Cognitive–Adaptive ( *n *= 82)					
Gestational age	−0.14	−0.03	0.8504	−0.30	0.24
Sex	−13.23	−0.43	<0.0001 *	−0.61	−0.25
Invasive procedure count	−0.10	−0.45	0.0049 *	−0.76	−0.14
Surgical history	−4.21	−0.13	0.3547	−0.42	0.15
Fentanyl exposure	8.21	0.16	0.1457	−0.06	0.37
Midazolam exposure	−0.04	−0.04	0.7124	−0.24	0.16
Parental visit frequency	−0.07	−0.11	0.2737	−0.32	0.09
Age during the examination	−0.29	−0.05	0.6199	−0.25	0.15
Maternal education	3.35	0.11	0.2443	−0.07	0.29
*R*^2^					0.4786
Adjusted *R*^2^					0.4106
Language–Social ( *n *= 82)					
Gestational age	0.71	0.12	0.4760	−0.21	0.44
Sex	−5.28	−0.16	0.1630	−0.38	0.06
Invasive procedure count	−0.06	−0.26	0.1640	−0.64	0.11
Surgical history	−5.24	−0.15	0.3860	−0.50	0.19
Fentanyl exposure	2.45	0.04	0.7420	−0.21	0.30
Midazolam exposure	0.06	0.05	0.7060	−0.20	0.29
Parental visit frequency	0.01	0.02	0.8630	−0.22	0.27
Age during the examination	−0.64	−0.10	0.4040	−0.34	0.14
Maternal education	3.67	0.11	0.3360	−0.11	0.32
*R*^2^					0.2497
Adjusted *R*^2^					0.1519

CI, confidence interval; * *p* < 0.00625 (Bonferroni-corrected significance threshold for eight comparisons). *B*, unstandardized regression coefficient; β, standardized regression coefficient. Only maternal education had missing values (*n* = 3).

## Data Availability

The data presented in this study are available on request from the corresponding author due to participant privacy and institutional ethical restrictions.

## Supplementary Materials

The following supporting information can be downloaded at https://www.mdpi.com/article/10.3390/children13081024/s1, Supplementary File S1: Sensitivity Analyses.

## Abbreviations

The following abbreviations are used in this manuscript:

CNS	Central nervous system
COVID-19	Coronavirus disease 2019
EMLA	Eutectic mixture of local anesthetics
NICU	Neonatal intensive care unit
STROBE	Strengthening the Reporting of Observational Studies in Epidemiology
GA	Gestational age
KSPD	Kyoto Scale of Psychological Development 2001
DQ	Developmental quotient
VIF	Variance inflation factor

## References

[1] Johnson S., Marlow N. (2011). Preterm Birth and Childhood Psychiatric Disorders. Pediatr. Res..

[2] Moore T., Hennessy E.M., Myles J., Johnson S.J., Draper E.S., Costeloe K.L., Marlow N. (2012). Neurological and Developmental Outcome in Extremely Preterm Children Born in England in 1995 and 2006. BMJ..

[3] Cruz M.D., Fernandes A.M., Oliveira C.R. (2016). Epidemiology of Painful Procedures Performed in Neonates: A Systematic Review of Observational Studies. Eur. J. Pain.

[4] Hathway G.J., Koch S., Low L., Fitzgerald M. (2009). The Changing Balance of Brainstem–spinal Cord Modulation of Pain Processing over the First Weeks of Rat Postnatal Life. J. Physiol..

[5] Boggini T., Pozzoli S., Schiavolin P., Erario R., Mosca F., Brambilla P., Fumagalli M. (2021). Cumulative Procedural Pain and Brain Development in Very Preterm Infants: A Systematic Review of Clinical and Preclinical Studies. Neurosci. Biobehav. Rev..

[6] Brummelte S., Grunau R.E., Chau V., Poskitt K.J., Brant R., Vinall J., Gover A., Synnes A.R., Miller S.P. (2012). Procedural Pain and Brain Development in Premature Newborns. Ann. Neurol..

[7] Ranger M., Chau C.M.Y., Garg A., Woodward T.S., Beg M.F., Bjornson B., Poskitt K., Fitzpatrick K., Synnes A.R., Miller S.P. (2013). Neonatal Pain-Related Stress Predicts Cortical Thickness at Age 7 Years in Children Born Very Preterm. PLoS ONE.

[8] Ranger M., Grunau R.E. (2014). Early Repetitive Pain in Preterm Infants in Relation to the Developing Brain. Pain Manag..

[9] Walker S.M. (2019). Long-term Effects of Neonatal Pain. Semin. Fetal Neonatal Med..

[10] Feldman R., Eidelman A.I., Sirota L., Weller A. (2003). Skin-to-Skin Contact (Kangaroo Care) Accelerates Autonomic and Neurobehavioural Maturation in Preterm Infants. Dev. Med. Child Neurol..

[11] Head L.M. (2014). The Effect of Kangaroo Care on Neurodevelopmental Outcomes in Preterm Infants. J. Perinat. Neonatal Nurs..

[12] Baudesson de Chanville A., Brevaut-Malaty V., Garbi A., Tosello B., Baumstarck K., Gire C. (2017). Analgesic Effect of Maternal Human Milk Odor on Premature Neonates: A Randomized Controlled Trial. J. Hum. Lact..

[13] Badiee Z., Asghari M. (2013). The Calming Effect of Maternal Breast Milk Odor on Premature Infants. Pediatr. Neonatol..

[14] Provenzi L., Broso S., Montirosso R. (2018). Do Mothers Sound Good? A Systematic Review of the Effects of Maternal Voice Exposure on Preterm Infants’ Development. Neurosci. Biobehav. Rev..

[15] Mann P.C., Stansfield B.K. (2024). Optimal Presence: Enhancing Parent Integration to Maximize Neurodevelopmental Outcomes in Preterm Infants. Pediatr. Res..

[16] Flacking R., Lehtonen L., Thomson G., Axelin A., Ahlqvist S., Moran V.H., Ewald U., Dykes F. (2012). Closeness and Separation in Neonatal Intensive Care. Acta. Paediatr..

[17] Welch M.G., Ludwig R.J. (2017). Calming Cycle Theory and the Co-regulation of Oxytocin. Psychodyn. Psychiatr..

[18] Johnson K. (2013). Maternal-infant Bonding: A Review of Literature. Int. J. Childbirth Edu..

[19] Cena L., Biban P., Janos J., Lavelli M., Langfus J., Tsai A., Youngstrom E.A., Stefana A. (2021). The Collateral Impact of COVID-19 Emergency on Neonatal Intensive Care Units and Family-Centered Care: Challenges and Opportunities. Front. Psychol..

[20] World Health Organization (2020). Clinical Management of COVID-19: Interim Guidance.

[21] Kostenzer J., von Rosenstiel-Pulver C., Hoffmann J., Walsh A., Mader S., Zimmermann L.J.I. (2022). Parents’ Experiences Regarding Neonatal Care During the COVID-19 Pandemic: Country-specific Findings of a Multinational Survey. BMJ Open.

[22] Ozawa M., Sakaki H., Meng X. (2021). Family Presence Restrictions and Telemedicine Use in Neonatal Intensive Care Units During the Coronavirus Disease Pandemic. Children.

[23] Kim H., Jang Y.H., Lee J.Y., Lee G.Y., Sung J.Y., Kim M.J., Lee B.G., Yang S., Kim J., Yoon K.S. (2024). Impact of COVID−19 Pandemic on Neurodevelopmental Outcome in Very Low Birth Weight Infants: A Nationwide Cohort Study. Front. Pediatr..

[24] Ohama N., Suga S., Watanabe S., Tanaka K., Kusuhara K. (2025). Neurodevelopmental Outcomes in Preterm Infants at 18 Months of Corrected Age Following Coronavirus Disease 2019 (COVID-19) Pandemic-Related Neonatal Intensive Care Unit (NICU) Care Changes. Cureus.

[25] Lau M., Kraus V., Schulze A.F., Rausch T.K., Krüger M., Göpel W. (2023). Observational Study on the Neonatal Outcome During the COVID-19 Pandemic in Germany. Acta. Paediatr..

[26] Ömercioğlu E., Mert Karakaya E.N., Özdemir G., Şencan Karakuş B., Kılınç Ş., İskender H.C., Cihan Çam E., Mete Yeşil A., Çelik H.T., Karahan S. (2024). Has the COVID-19 Pandemic Negatively Impacted Children’s Development? an Assessment of the Neurodevelopment of Premature Babies Born During the Pandemic. Turk. J. Pediatr..

[27] Tseng T.-C., Wang T.-M., Hsu Y.-C., Hsu C.-T., Lin Y.-H., Lin M.-C. (2024). Impact of COVID-19 Pandemic on Neurodevelopmental Outcomes of Premature Infants: A Retrospective National Cohort Study. BMJ Paediatr. Open..

[28] von Elm E., Altman D.G., Egger M., Pocock S.J., Gøtzsche P.C., Vandenbroucke J.P. (2008). The Strengthening the Reporting of Observational Studies in Epidemiology (STROBE) Statement: Guidelines for Reporting Observational Studies. J. Clin. Epidemiol..

[29] Inder T.E., Volpe J.J., Anderson P.J. (2023). Defining the Neurologic Consequences of Preterm Birth. N. Engl. J. Med..

[30] Fan X., Wu N., Tu Y., Zang T., Bai J., Peng G., Liu Y. (2024). Perinatal Depression and Infant and Toddler Neurodevelopment: A Systematic Review and Meta-Analysis. Neurosci. Biobehav. Rev..

[31] Grunau R.E., Whitfield M.F., Petrie-Thomas J., Synnes A.R., Cepeda I.L., Keidar A., Rogers M., MacKay M., Hubber-Richard P., Johannesen D. (2009). Neonatal Pain, Parenting Stress and Interaction, in Relation to Cognitive and Motor Development at 8 and 18 Months in Preterm Infants. Pain.

[32] Scala M., Marchman V.A., Brignoni-Pérez E., Chan Morales M., Dubner S.E., Travis K.E. (2021). Impact of the COVID-19 Pandemic on Developmental Care Practices for Infants Born Preterm. Early Hum. Dev..

[33] Cuartas J. (2022). The Effect of Maternal Education on Parenting and Early Childhood Development: An Instrumental Variables Approach. J. Fam. Psychol..

[34] Ikuzawa M., Matsushita Y., Nakase A. (2002). Shinpan K Shiki Hattatsu Kensahou 2001 Nenban (The Kyoto Scale of Psychological Development Test).

[35] Kono Y., Yonemoto N., Kusuda S., Hirano S., Iwata O., Tanaka K., Nakazawa J. (2016). Developmental Assessment of VLBW Infants at 18 months of Age: A Comparison Study Between KSPD and Bayley III. Brain Dev..

[36] Tortora D., Severino M., Biase C.D., Malova M., Parodi A., Minghetti D., Traggiai C., Uccella S., Boeri L., Morana G. (2019). Early Pain Exposure Influences Functional Brain Connectivity in Very Preterm Neonates. Front. Neurosci..

[37] Duerden E.G., Grunau R.E., Chau V., Groenendaal F., Guo T., Chakravarty M.M., Benders M., Wagenaar N., Eijsermans R., Koopman C. (2020). Association of Early Skin Breaks and Neonatal Thalamic Maturation: A Modifiable Risk?. Neurology.

[38] Ing M.C., Keane O.A., Lakshmanan A., Kim E., Lee H.C., Kelley-Quon L.I. (2024). Opioid Equipotency Conversions for Hospitalized Infants: A Systematic Review. J. Perinatol..

[39] Giordano V., Deindl P., Gal E., Unterasinger L., Fuiko R., Steinbauer P., Weninger M., Berger A., Olischar M. (2023). Pain and Neurodevelopmental Outcomes of Infants Born Very Preterm. Dev. Med. Child. Neurol..

[40] Schneider J., Duerden E.G., Guo T., Ng K., Hagmann P., Bickle Graz M., Grunau R.E., Chakravarty M.M., Hüppi P.S., Truttmann A.C. (2018). Procedural Pain and Oral Glucose in Preterm Neonates: Brain Development and Sex-Specific Effects. Pain.

[41] Vinall J., Miller S.P., Synnes A.R., Grunau R.E. (2013). Parent Behaviors Moderate the Relationship between Neonatal Pain and Internalizing Behaviors at 18 Months of Corrected Age in Children Born Very Prematurely. Pain.

[42] Zwicker J.G., Miller S.P., Grunau R.E., Chau V., Brant R., Studholme C., Liu M., Synnes A., Poskitt K.J., Stiver M.L. (2016). Smaller Cerebellar Growth and Poorer Neurodevelopmental Outcomes in Very Preterm Infants Exposed to Neonatal Morphine. J. Pediatr..

[43] McLean M.A., Ranger M., Bone J.N., Selvanathan T., Au-Young S.H., Chau C.M.Y., Chau V., Ly L., Kelly E., Synnes A. (2025). Neonatal Sucrose and Internalizing Behaviors at 18 Months in Children Born Very Preterm. JAMA Netw. Open.

[44] Thompson D.K., Lee K.J., Egan G.F., Warfield S.K., Doyle L.W., Anderson P.J., Inder T.E. (2014). Regional White Matter Microstructure in Very Preterm Infants: Predictors and 7-Year Outcomes. Cortex.

[45] Cong X., Wu J., Vittner D., Xu W., Hussain N., Galvin S., Fitzsimons M., McGrath J.M., Henderson W.A. (2017). The Impact of Cumulative Pain/Stress on Neurobehavioral Development of Preterm Infants in the NICU. Early Hum. Dev..

[46] Bhutta A.T., Cleves M.A., Casey P.H., Cradock M.M., Anand K.J.S. (2002). Cognitive and Behavioral Outcomes of School-Aged Children Who Were Born Preterm: A Meta-Analysis. JAMA.

[47] Marlow N., Wolke D., Bracewell M.A., Samara M., EPICure Study Group (2005). Neurologic and Developmental Disability at Six Years of Age after Extremely Preterm Birth. N. Engl. J. Med..

[48] Montirosso R., Casini E., Del Prete A., Zanini R., Bellù R., Borgatti R. (2016). Neonatal Developmental Care in Infant Pain Management and Internalizing Behaviors at 18 Months in Prematurely Born Children. Eur. J. Pain.

[49] Tessier R., Cristo M.B., Velez S., Giron M., Nadeau L., Figueroa de Calume Z., Ruiz-Paláez J.G., Charpak N. (2003). Kangaroo Mother Care: A Method for Protecting High-risk Low-birth-weight and Premature Infants Against Developmental Delay. Infant Behav. Dev..

[50] Lazarus M.F., Marchman V.A., Brignoni-Pérez E., Dubner S., Feldman H.M., Scala M., Travis K.E. (2024). Inpatient Skin-to-skin Care Predicts 12-month Neurodevelopmental Outcomes in Very Preterm Infants. J. Pediatr..

[51] Kristoffersen L., Støen R., Bergseng H., Flottorp S.T., Magerøy G., Grunewaldt K.H., Aker K. (2025). Immediate Skin-to-skin Contact in Very Preterm Neonates and Early Childhood Neurodevelopment: A Randomized Clinical Trial. JAMA Netw. Open..

[52] Lode-Kolz K., Jonas W., Hetland H.B., Hovland Instebø K.H., Tokvam H., Pike H., Lilliesköld S., Klemming S., Linnér A., Ådén U. (2025). Immediate Skin-to-skin Contact at Very Preterm Birth and Neurodevelopment the First Two Years: Secondary Outcomes from a Randomised Clinical Trial. Children.

[53] Sato K., Fukai T., Fujisawa K.K., Nakamuro M. (2023). Association Between the COVID-19 Pandemic and Early Childhood Development. JAMA Pediatr..

