# Supplementary material for: Association Between Neonatal Pain Exposure and Neurodevelopmental Outcomes in Very Preterm Infants During the COVID-19 Pandemic: A Retrospective Observational Study
Source: Children (Basel). 2026 Aug 1;13(8):1024. doi: 10.3390/children13081024 (PMC13510993; doi:10.3390/children13081024)
Supplement: Supplementary file 1 [file children-13-01024-s001.zip › children-4433579-Supplemental file.pdf]

Supplementary File S1. Sensitivity Analyses

Table S1. Sensitivity analysis: Results of multiple regression analysis on the developmental quotient in the corrected 18-month Kyoto Scale of Psychological Development in infants with a birth weight <1500 g (n = 84).

	Overall					Postural–Motor					Cognitive–Adaptive					Language–Social				
	B	β	p value	95%CI		B	β	p value	95%CI		B	β	p value	95%CI		B	β	p value	95%CI	
Gestational age	-0.88	-0.16	0.3396	-0.48	0.17	-1.08	-0.15	0.4062	-0.52	0.21	-0.79	-0.14	0.4198	-0.47	0.20	-0.48	-0.08	0.6384	-0.41	0.25
Sex	-3.00	-0.10	0.3793	-0.32	0.12	-5.83	-0.15	0.2246	-0.40	0.10	-1.63	-0.05	0.6509	-0.28	0.18	-4.84	-0.15	0.2035	-0.37	0.08
Invasive procedure count	-0.09	-0.40	0.0224	-0.74	-0.06	-0.11	-0.37	0.0637	-0.75	0.02	-0.09	-0.38	0.0351	-0.74	-0.03	-0.09	-0.37	0.0387	-0.72	-0.02
Surgical history	-4.05	-0.13	0.4249	-0.45	0.19	-7.35	-0.19	0.3032	-0.55	0.17	-3.87	-0.12	0.4718	-0.45	0.21	1.49	0.04	0.7910	-0.28	0.37
Fentanyl exposure	-0.15	0.00	0.9873	-0.27	0.26	-5.56	-0.06	0.6671	-0.36	0.23	4.16	0.06	0.6698	-0.21	0.33	-6.66	-0.09	0.5153	-0.36	0.18
Midazolam exposure	0.14	0.11	0.3488	-0.12	0.33	0.05	0.03	0.8186	-0.23	0.28	0.12	0.09	0.4408	-0.14	0.32	0.16	0.12	0.3115	-0.11	0.35
Parental visit frequency	-0.02	-0.03	0.7692	-0.27	0.20	0.04	0.05	0.7133	-0.22	0.32	-0.02	-0.04	0.7327	-0.29	0.20	-0.01	-0.02	0.8800	-0.26	0.23
Age during the examination	-0.10	-0.01	0.9330	-0.23	0.21	3.32	0.24	0.0580	-0.01	0.49	0.02	0.00	0.9897	-0.23	0.23	-0.85	-0.07	0.5354	-0.30	0.16
Maternal education	-2.38	-0.07	0.4910	-0.29	0.14	1.35	0.03	0.7805	-0.21	0.28	-3.58	-0.11	0.3302	-0.33	0.11	-0.85	-0.02	0.8244	-0.25	0.20
R^2					0.2446					0.2655					0.2135					0.1892
Adjusted R^2					0.1332					0.1571					0.0974					0.0696

CI, confidence interval; B, unstandardized regression coefficient; β, standardized regression coefficient. Bonferroni-corrected significance threshold: $p < 0.00625$.

Table S2. Sensitivity analysis: Results of multiple regression analysis on the developmental quotient in the 36-month Kyoto Scale of Psychological Development in infants with a birth weight <1500 g (n = 75).

	Overall					Postural–Motor					Cognitive–Adaptive					Language–Social				
	B	β	p value	95%CI		B	β	p value	95%CI		B	β	p value	95%CI		B	β	p value	95%CI	
Gestational age	-0.17	-0.03	0.8394	-0.34	0.28	-0.84	-0.10	0.5452	-0.45	0.24	-0.41	-0.07	0.6235	-0.37	0.22	0.13	0.02	0.9094	-0.33	0.37
Sex	-9.32	-0.31	0.0038*	-0.52	-0.10	-8.31	-0.19	0.1033	-0.42	0.04	-13.06	-0.43	0.0001*	-0.63	-0.23	-5.94	-0.17	0.1475	-0.41	0.06
Invasive procedure count	-0.10	-0.45	0.0090	-0.79	-0.12	-0.10	-0.30	0.1123	-0.67	0.07	-0.12	-0.52	0.0021*	-0.84	-0.20	-0.08	-0.33	0.0914	-0.71	0.05
Surgical history	-5.91	-0.19	0.2129	-0.50	0.11	-13.39	-0.30	0.0817	-0.64	0.04	-4.60	-0.15	0.3165	-0.44	0.15	-5.96	-0.17	0.3353	-0.52	0.18
Fentanyl exposure	4.82	0.10	0.4073	-0.14	0.33	-2.61	-0.04	0.7795	-0.29	0.22	9.03	0.18	0.1116	-0.04	0.40	3.26	0.06	0.6677	-0.21	0.32
Midazolam exposure	0.00	0.00	0.9805	-0.22	0.21	0.07	0.04	0.7344	-0.20	0.28	-0.07	-0.06	0.5830	-0.26	0.15	0.04	0.03	0.8193	-0.22	0.27
Parental visit frequency	-0.02	-0.04	0.7358	-0.26	0.19	0.00	0.01	0.9653	-0.24	0.25	-0.06	-0.11	0.2999	-0.33	0.10	0.01	0.01	0.9243	-0.24	0.27
Age during the examination	-0.22	-0.04	0.7125	-0.23	0.16	0.80	0.09	0.4137	-0.13	0.30	-0.07	-0.01	0.9083	-0.20	0.18	-0.42	-0.06	0.5990	-0.28	0.16
Maternal education	4.00	0.13	0.1988	-0.07	0.33	-1.22	-0.03	0.8075	-0.24	0.19	3.86	0.12	0.2014	-0.07	0.31	4.61	0.13	0.2572	-0.10	0.35
R^2					0.4260					0.2983					0.4940					0.2582
Adjusted R^2					0.3440					0.1964					0.4217					0.1522

CI, confidence interval; *$p < 0.00625$ (Bonferroni-corrected significance threshold for eight comparisons). B, unstandardized regression coefficient; β, standardized regression coefficient

Table S3. Sensitivity analysis: Results of multiple regression analysis on the developmental quotient in the corrected 18-month Kyoto Scale of Psychological Development after additional adjustment for duration of mechanical ventilation (n = 92).

	Overall					Postural–Motor					Cognitive–Adaptive					Language–Social				
	B	β	p value	95%CI		B	β	p value	95%CI		B	β	p value	95%CI		B	β	p value	95%CI	
Gestational age	-2.20	-0.41	0.0439	-0.80	-0.01	-2.99	-0.42	0.0518	-0.85	0.00	-1.89	-0.34	0.1080	-0.75	0.08	-2.43	-0.41	0.0385	-0.81	-0.02
Sex	-4.25	-0.14	0.1887	-0.35	0.07	-7.73	-0.20	0.0918	-0.42	0.03	-2.96	-0.09	0.3970	-0.32	0.13	-6.04	-0.18	0.0840	-0.39	0.03
Invasive procedure count	-0.05	-0.20	0.2738	-0.57	0.16	-0.05	-0.17	0.3885	-0.57	0.22	-0.06	-0.24	0.2150	-0.63	0.14	-0.02	-0.06	0.7295	-0.43	0.30
Surgical history	-1.18	-0.04	0.8129	-0.36	0.28	-3.97	-0.10	0.5719	-0.44	0.25	-1.66	-0.05	0.7590	-0.39	0.28	6.01	0.18	0.2632	-0.14	0.49
Fentanyl exposure	4.61	0.07	0.6107	-0.20	0.33	1.22	0.01	0.9236	-0.27	0.30	7.68	0.11	0.4350	-0.17	0.39	0.87	0.01	0.9291	-0.25	0.27
Midazolam exposure	0.18	0.21	0.1828	-0.10	0.53	0.08	0.07	0.6724	-0.27	0.42	0.16	0.18	0.2950	-0.16	0.51	0.25	0.26	0.1003	-0.05	0.58
Parental visit frequency	-0.06	-0.11	0.3932	-0.35	0.14	-0.01	-0.02	0.8852	-0.28	0.25	-0.06	-0.10	0.4230	-0.36	0.15	-0.06	-0.11	0.3719	-0.35	0.13
Age during the examination	0.31	0.03	0.7801	-0.19	0.26	3.59	0.28	0.0270	0.03	0.52	0.37	0.04	0.7600	-0.20	0.27	-0.33	-0.03	0.7822	-0.26	0.19
Maternal education	-2.18	-0.07	0.5099	-0.28	0.14	1.54	0.04	0.7409	-0.19	0.26	-3.47	-0.11	0.3340	-0.32	0.11	-0.40	-0.01	0.9102	-0.22	0.19
Duration of mechanical ventilation	-0.35	-0.54	0.0371	-1.04	-0.03	-0.46	-0.54	0.0517	-1.08	0.00	-0.26	-0.39	0.1440	-0.92	0.14	-0.56	-0.80	0.0020*	-1.30	-0.30
R^2					0.2910					0.3082					0.2384					0.2984
Adjusted R^2					0.1785					0.1984					0.1175					0.1870

CI, confidence interval; B, unstandardized regression coefficient; β, standardized regression coefficient. Bonferroni-corrected significance threshold: $p < 0.00625$.

Table S4. Sensitivity analysis: Results of multiple regression analysis on the developmental quotient in the 36-month Kyoto Scale of Psychological Development after additional adjustment for duration of mechanical ventilation (n = 82).

	Overall					Postural–Motor					Cognitive–Adaptive					Language–Social				
	B	β	p value	95%CI		B	β	p value	95%CI		B	β	p value	95%CI		B	β	p value	95%CI	
Gestational age	-1.16	-0.22	0.2456	-0.59	0.15	-0.66	-0.08	0.6770	-0.48	0.31	-1.66	-0.30	0.0861	-0.65	0.04	-0.73	-0.12	0.5769	-0.55	0.31
Sex	-10.44	-0.35	0.0006*	-0.54	-0.16	-7.88	-0.18	0.0943	-0.38	0.03	-14.62	-0.48	<0.0001*	-0.66	-0.30	-6.59	-0.19	0.0856	-0.42	0.03
Invasive procedure count	-0.05	-0.22	0.2298	-0.58	0.14	-0.10	-0.32	0.1032	-0.71	0.07	-0.06	-0.26	0.1361	-0.60	0.08	-0.02	-0.10	0.6464	-0.51	0.32
Surgical history	-3.06	-0.10	0.5134	-0.40	0.20	-13.98	-0.31	0.0655	-0.63	0.02	-1.61	-0.05	0.7216	-0.34	0.23	-2.78	-0.08	0.6501	-0.43	0.27
Fentanyl exposure	6.98	0.14	0.2299	-0.09	0.36	-2.98	-0.04	0.7466	-0.28	0.20	11.48	0.22	0.0425	0.01	0.43	5.54	0.10	0.4650	-0.16	0.36
Midazolam exposure	0.06	0.05	0.6300	-0.16	0.26	0.06	0.03	0.7646	-0.19	0.26	0.01	0.01	0.9495	-0.19	0.20	0.11	0.08	0.4964	-0.16	0.33
Parental visit frequency	-0.06	-0.10	0.3812	-0.31	0.12	0.01	0.01	0.9151	-0.22	0.25	-0.10	-0.18	0.0894	-0.38	0.03	-0.02	-0.03	0.7954	-0.28	0.22
Age during the examination	-0.27	-0.05	0.6476	-0.26	0.16	0.74	0.09	0.4270	-0.13	0.31	-0.10	-0.02	0.8659	-0.21	0.18	-0.46	-0.07	0.5467	-0.31	0.17
Maternal education	2.92	0.10	0.3107	-0.09	0.28	-0.88	-0.02	0.8475	-0.22	0.18	3.05	0.10	0.2725	-0.08	0.27	3.39	0.10	0.3685	-0.12	0.31
Duration of mechanical ventilation	-0.32	-0.50	0.0423	-0.98	-0.02	0.06	0.06	0.8097	-0.45	0.57	-0.36	-0.55	0.0178	-1.00	-0.10	-0.34	-0.47	0.0961	-1.02	0.09
R^2					0.4464					0.3195					0.5202					0.2799
Adjusted R^2					0.3650					0.2179					0.4496					0.1740

CI, confidence interval; *p < 0.00625 (Bonferroni-corrected significance threshold for eight comparisons). B, unstandardized regression coefficient; β, standardized regression coefficient

Table S5. Sensitivity analysis: Results of multiple regression analysis on the developmental quotient in the corrected 18-month Kyoto Scale of Psychological Development after additional adjustment for NICU stay duration (n = 92).

	Overall					Postural–Motor					Cognitive–Adaptive					Language–Social				
	B	β	p value	95%CI		B	β	p value	95%CI		B	β	p value	95%CI		B	β	p value	95%CI	
Gestational age	-0.85	-0.16	0.3169	-0.47	0.15	-1.29	-0.18	0.2727	-0.51	0.15	-1.89	-0.34	0.1080	-0.75	0.08	-0.88	-0.16	0.3289	-0.48	0.16
Sex	-2.08	-0.07	0.5244	-0.28	0.15	-4.38	-0.11	0.3344	-0.34	0.12	-2.96	-0.09	0.3970	-0.32	0.13	-1.23	-0.04	0.7242	-0.26	0.18
Invasive procedure count	-0.01	-0.04	0.8893	-0.64	0.56	0.05	0.15	0.6303	-0.48	0.79	-0.06	-0.24	0.2150	-0.63	0.14	-0.02	-0.09	0.7849	-0.71	0.54
Surgical history	-3.00	-0.10	0.5449	-0.41	0.22	-5.89	-0.14	0.3921	-0.48	0.19	-1.66	-0.05	0.7590	-0.39	0.28	-2.94	-0.09	0.5794	-0.42	0.24
Fentanyl exposure	5.37	0.08	0.5860	-0.21	0.37	5.74	0.06	0.6743	-0.24	0.37	7.68	0.11	0.4350	-0.17	0.39	8.90	0.13	0.3999	-0.17	0.42
Midazolam exposure	0.13	0.16	0.3372	-0.17	0.48	0.01	0.01	0.9688	-0.34	0.35	0.16	0.18	0.2950	-0.16	0.51	0.12	0.13	0.4331	-0.20	0.47
Parental visit frequency	-0.04	-0.07	0.5948	-0.31	0.18	0.00	0.00	0.9743	-0.26	0.27	-0.06	-0.10	0.4230	-0.36	0.15	-0.04	-0.08	0.5381	-0.33	0.18
Age during the examination	0.11	0.01	0.9243	-0.22	0.24	3.29	0.25	0.0416	0.01	0.50	0.37	0.04	0.7600	-0.20	0.27	0.21	0.02	0.8612	-0.22	0.26
Maternal education	-1.56	-0.05	0.6471	-0.27	0.17	2.85	0.07	0.5482	-0.16	0.30	-3.47	-0.11	0.3340	-0.32	0.11	-2.91	-0.09	0.4269	-0.31	0.13
NICU stay duration	-0.15	-0.44	0.1793	-1.08	0.21	-0.28	-0.65	0.0611	-1.33	0.03	-0.26	-0.39	0.1440	-0.92	0.14	-0.13	-0.37	0.2736	-1.03	0.30
R^2					0.2616					0.3051					0.2384					0.2269
Adjusted R^2					0.1444					0.1948					0.1175					0.1042

CI, confidence interval; B, unstandardized regression coefficient; β, standardized regression coefficient. Bonferroni-corrected significance threshold: $p < 0.00625$.

Table S6. Sensitivity analysis: Results of multiple regression analysis on the developmental quotient in the 36-month Kyoto Scale of Psychological Development after additional adjustment for NICU stay duration (n = 82).

	Overall					Postural–Motor					Cognitive–Adaptive					Language–Social				
	B	β	p value	95%CI		B	β	p value	95%CI		B	β	p value	95%CI		B	β	p value	95%CI	
Gestational age	0.17	0.03	0.8262	-0.26	0.32	-0.98	-0.12	0.4196	-0.43	0.18	-0.20	-0.04	0.7873	-0.31	0.24	0.77	0.13	0.4482	-0.20	0.46
Sex	-9.20	-0.31	0.0024*	-0.50	-0.11	-8.06	-0.18	0.0804	-0.38	0.02	-13.17	-0.43	<0.0001*	-0.61	-0.24	-5.33	-0.16	0.1620	-0.38	0.06
Invasive procedure count	-0.08	-0.37	0.2173	-0.95	0.22	-0.07	-0.20	0.5098	-0.81	0.41	-0.07	-0.31	0.2643	-0.87	0.24	-0.09	-0.38	0.2619	-1.05	0.29
Surgical history	-5.30	-0.17	0.2633	-0.48	0.13	-13.23	-0.29	0.0741	-0.61	0.03	-3.91	-0.12	0.3951	-0.41	0.17	-5.51	-0.16	0.3676	-0.51	0.19
Fentanyl exposure	4.43	0.09	0.4885	-0.16	0.34	-0.80	-0.01	0.9355	-0.27	0.25	9.80	0.19	0.1181	-0.05	0.42	0.99	0.02	0.9051	-0.27	0.30
Midazolam exposure	0.01	0.01	0.9198	-0.20	0.22	0.06	0.04	0.7409	-0.18	0.26	-0.05	-0.04	0.6930	-0.24	0.16	0.06	0.05	0.6933	-0.19	0.29
Parental visit frequency	-0.02	-0.04	0.7080	-0.27	0.18	0.00	-0.01	0.9616	-0.24	0.23	-0.08	-0.13	0.2287	-0.34	0.08	0.02	0.03	0.7872	-0.22	0.29
Age during the examination	-0.44	-0.08	0.4602	-0.29	0.13	0.74	0.09	0.4238	-0.13	0.31	-0.32	-0.06	0.5874	-0.26	0.15	-0.61	-0.10	0.4283	-0.34	0.15
Maternal education	3.23	0.11	0.2801	-0.09	0.30	-0.70	-0.02	0.8797	-0.22	0.18	3.57	0.11	0.2211	-0.07	0.30	3.47	0.10	0.3695	-0.12	0.32
NICU stay duration	-0.01	-0.04	0.8975	-0.65	0.57	-0.06	-0.13	0.6940	-0.76	0.51	-0.06	-0.18	0.5471	-0.75	0.40	0.05	0.15	0.6772	-0.55	0.84
R^2					0.4117					0.3205					0.4814					0.2517
Adjusted R^2					0.3252					0.2191					0.4051					0.1416

CI, confidence interval; *p < 0.00625 (Bonferroni-corrected significance threshold for eight comparisons). B, unstandardized regression coefficient; β, standardized regression coefficient

Table S7. Sensitivity analysis: Results of multiple regression analysis on the developmental quotient in the corrected 18-month Kyoto Scale of Psychological Development after additional adjustment for birth weight (n = 92).

	Overall					Postural–Motor					Cognitive–Adaptive					Language–Social				
	B	β	p value	95%CI		B	β	p value	95%CI		B	β	p value	95%CI		B	β	p value	95%CI	
Gestational age	-1.52	-0.28	0.1308	-0.65	0.09	-1.77	-0.25	0.2121	-0.65	0.15	-1.35	-0.24	0.2092	-0.63	0.14	-0.80	-0.14	0.4748	-0.52	0.24
Sex	-3.67	-0.12	0.2614	-0.34	0.09	-6.64	-0.17	0.1536	-0.40	0.06	-2.50	-0.08	0.4757	-0.30	0.14	-4.56	-0.14	0.2154	-0.36	0.08
Invasive procedure count	-0.08	-0.34	0.0456	-0.68	-0.01	-0.10	-0.33	0.0787	-0.69	0.04	-0.08	-0.34	0.0529	-0.69	0.00	-0.07	-0.30	0.0919	-0.65	0.05
Surgical history	-3.59	-0.12	0.4653	-0.43	0.20	-7.21	-0.18	0.3024	-0.52	0.16	-3.48	-0.11	0.5103	-0.44	0.22	1.97	0.06	0.7211	-0.27	0.38
Fentanyl exposure	-0.80	-0.01	0.9282	-0.27	0.25	-5.65	-0.06	0.6564	-0.35	0.22	3.62	0.05	0.7069	-0.22	0.32	-7.53	-0.10	0.4550	-0.37	0.17
Midazolam exposure	0.12	0.14	0.4018	-0.19	0.46	0.01	0.01	0.9700	-0.35	0.36	0.11	0.12	0.4767	-0.22	0.46	0.16	0.17	0.3194	-0.17	0.51
Parental visit frequency	-0.02	-0.04	0.7203	-0.28	0.20	0.03	0.05	0.7185	-0.21	0.31	-0.03	-0.06	0.6436	-0.31	0.19	-0.01	-0.01	0.9445	-0.26	0.24
Age during the examination	-0.14	-0.01	0.9013	-0.25	0.22	3.10	0.24	0.0632	-0.01	0.49	0.04	0.00	0.9762	-0.24	0.25	-0.89	-0.08	0.4981	-0.32	0.16
Maternal education	-1.99	-0.06	0.5548	-0.28	0.15	1.63	0.04	0.7326	-0.19	0.27	-3.34	-0.10	0.3581	-0.32	0.12	-0.34	-0.01	0.9280	-0.23	0.21
Birth weight	0.01	0.26	0.1490	-0.10	0.61	0.01	0.18	0.3570	-0.21	0.56	0.01	0.18	0.3297	-0.19	0.55	0.01	0.23	0.2186	-0.14	0.59
R^2					0.2649					0.2749					0.2238					0.2026
Adjusted R^2					0.1482					0.1598					0.1006					0.0761

CI, confidence interval; B, unstandardized regression coefficient; β, standardized regression coefficient. Bonferroni-corrected significance threshold: $p < 0.00625$.

Table S8. Sensitivity analysis: Results of multiple regression analysis on the developmental quotient in the 36-month Kyoto Scale of Psychological Development after additional adjustment for birth weight (n = 82).

	Overall					Postural–Motor					Cognitive–Adaptive					Language–Social				
	B	β	p value	95%CI		B	β	p value	95%CI		B	β	p value	95%CI		B	β	p value	95%CI	
Gestational age	-0.50	-0.09	0.6089	-0.46	0.27	-1.69	-0.21	0.2672	-0.59	0.17	-0.92	-0.17	0.3305	-0.51	0.17	0.23	0.04	0.8586	-0.38	0.45
Sex	-10.12	-0.34	0.0012*	-0.54	-0.14	-9.14	-0.20	0.0552	-0.41	0.00	-14.27	-0.46	<0.0001*	-0.65	-0.28	-5.92	-0.17	0.1342	-0.40	0.06
Invasive procedure count	-0.08	-0.36	0.0341	-0.69	-0.03	-0.09	-0.27	0.1258	-0.62	0.08	-0.09	-0.41	0.0112	-0.73	-0.10	-0.06	-0.24	0.2140	-0.62	0.14
Surgical history	-5.50	-0.18	0.2387	-0.48	0.12	-13.72	-0.30	0.0616	-0.61	0.01	-4.36	-0.14	0.3348	-0.42	0.15	-5.33	-0.15	0.3802	-0.50	0.19
Fentanyl exposure	3.20	0.06	0.5799	-0.16	0.29	-3.44	-0.05	0.7019	-0.28	0.19	7.20	0.14	0.2026	-0.08	0.35	1.83	0.03	0.8085	-0.23	0.29
Midazolam exposure	0.00	0.00	0.9840	-0.21	0.21	0.06	0.03	0.7755	-0.19	0.25	-0.06	-0.05	0.6366	-0.25	0.15	0.05	0.04	0.7432	-0.20	0.28
Parental visit frequency	-0.03	-0.05	0.6779	-0.26	0.17	0.00	0.00	0.9980	-0.23	0.23	-0.07	-0.12	0.2429	-0.32	0.08	0.01	0.02	0.8888	-0.23	0.26
Age during the examination	-0.34	-0.06	0.5657	-0.28	0.15	0.88	0.11	0.3440	-0.12	0.33	-0.18	-0.03	0.7566	-0.23	0.17	-0.58	-0.09	0.4598	-0.34	0.15
Maternal education	3.86	0.13	0.1996	-0.07	0.32	-0.17	0.00	0.9707	-0.21	0.20	4.12	0.13	0.1591	-0.05	0.31	4.15	0.12	0.2898	-0.10	0.34
Birth weight	0.01	0.18	0.2585	-0.13	0.49	0.01	0.14	0.4056	-0.19	0.46	0.01	0.20	0.1840	-0.10	0.50	0.00	0.11	0.5401	-0.25	0.47
R^2					0.4226					0.3260					0.4920					0.2539
Adjusted R^2					0.3377					0.2254					0.4173					0.1442

CI, confidence interval; *p < 0.00625 (Bonferroni-corrected significance threshold for eight comparisons). B, unstandardized regression coefficient; β, standardized regression coefficient

Table S9. Sensitivity analysis: Results of multiple regression analysis on the developmental quotient in the corrected 18-month Kyoto Scale of Psychological Development after additional adjustment for the 5-minute Apgar score (n = 92).

	Overall					Postural–Motor					Cognitive–Adaptive					Language–Social				
	B	β	p value	95%CI		B	β	p value	95%CI		B	β	p value	95%CI		B	β	p value	95%CI	
Gestational age	-0.78	-0.14	0.3749	-0.47	0.18	-1.40	-0.20	0.2463	-0.54	0.14	-0.74	-0.13	0.4251	-0.47	0.20	-0.17	-0.03	0.8633	-0.36	0.31
Sex	-2.49	-0.08	0.4625	-0.30	0.14	-5.99	-0.15	0.1980	-0.38	0.08	-1.26	-0.04	0.7261	-0.27	0.19	-3.85	-0.12	0.3115	-0.35	0.11
Invasive procedure count	-0.08	-0.36	0.0427	-0.71	-0.01	-0.09	-0.30	0.1064	-0.66	0.07	-0.08	-0.36	0.0501	-0.71	0.00	-0.08	-0.32	0.0840	-0.68	0.04
Surgical history	-4.09	-0.13	0.4219	-0.46	0.19	-7.86	-0.19	0.2609	-0.53	0.15	-3.97	-0.12	0.4624	-0.46	0.21	1.65	0.05	0.7718	-0.29	0.39
Fentanyl exposure	0.20	0.00	0.9823	-0.27	0.27	-4.13	-0.05	0.7428	-0.33	0.23	4.41	0.06	0.6516	-0.21	0.34	-6.67	-0.09	0.5186	-0.37	0.19
Midazolam exposure	0.16	0.18	0.2771	-0.15	0.52	0.07	0.06	0.7254	-0.29	0.41	0.13	0.15	0.3874	-0.19	0.49	0.20	0.21	0.2226	-0.13	0.56
Parental visit frequency	-0.01	-0.03	0.8309	-0.28	0.22	0.04	0.05	0.6950	-0.21	0.31	-0.02	-0.04	0.7619	-0.29	0.22	0.00	0.00	0.9857	-0.26	0.25
Age during the examination	0.24	0.02	0.8418	-0.22	0.27	3.95	0.30	0.0187	0.05	0.56	0.21	0.02	0.8660	-0.23	0.27	-0.43	-0.04	0.7479	-0.29	0.21
Maternal education	-2.34	-0.07	0.4997	-0.29	0.14	1.07	0.03	0.8208	-0.20	0.25	-3.53	-0.11	0.3384	-0.33	0.12	-0.73	-0.02	0.8511	-0.25	0.20
5-minute Apgar score	0.63	0.08	0.5291	-0.16	0.31	1.85	0.17	0.1799	-0.08	0.42	0.45	0.05	0.6696	-0.19	0.30	0.54	0.06	0.6342	-0.19	0.31
R^2					0.2451					0.2905					0.2173					0.1837
Adjusted R^2					0.1214					0.1742					0.0890					0.0499

CI, confidence interval; B, unstandardized regression coefficient; β, standardized regression coefficient. Bonferroni-corrected significance threshold: $p < 0.00625$.

Table S10. Sensitivity analysis: Results of multiple regression analysis on the developmental quotient in the 36-month Kyoto Scale of Psychological Development after additional adjustment for the 5-minute Apgar score (n = 82).

	Overall					Postural–Motor					Cognitive–Adaptive					Language–Social				
	B	β	p value	95%CI		B	β	p value	95%CI		B	β	p value	95%CI		B	β	p value	95%CI	
Gestational age	0.13	0.02	0.8674	-0.27	0.32	-1.12	-0.14	0.3579	-0.44	0.16	-0.14	-0.03	0.8524	-0.31	0.25	0.63	0.10	0.5360	-0.23	0.44
Sex	-9.14	-0.31	0.0037*	-0.51	-0.10	-7.11	-0.16	0.1340	-0.37	0.05	-13.29	-0.43	<0.0001*	-0.62	-0.24	-5.36	-0.16	0.1780	-0.39	0.07
Invasive procedure count	-0.08	-0.39	0.0262	-0.73	-0.05	-0.08	-0.26	0.1487	-0.61	0.09	-0.10	-0.46	0.0058*	-0.78	-0.14	-0.06	-0.25	0.1940	-0.64	0.13
Surgical history	-5.46	-0.18	0.2554	-0.49	0.13	-14.36	-0.31	0.0532	-0.63	0.00	-4.20	-0.13	0.3678	-0.43	0.16	-5.25	-0.15	0.3970	-0.51	0.20
Fentanyl exposure	4.53	0.09	0.4534	-0.15	0.32	0.43	0.01	0.9626	-0.23	0.25	7.96	0.15	0.1781	-0.07	0.37	2.84	0.05	0.7170	-0.22	0.32
Midazolam exposure	0.02	0.01	0.8919	-0.20	0.23	0.09	0.05	0.6586	-0.17	0.27	-0.05	-0.04	0.7116	-0.24	0.17	0.07	0.05	0.6890	-0.20	0.30
Parental visit frequency	-0.02	-0.04	0.7045	-0.26	0.18	0.00	0.00	0.9917	-0.23	0.23	-0.07	-0.12	0.2751	-0.32	0.09	0.01	0.02	0.9020	-0.24	0.27
Age during the examination	-0.47	-0.08	0.4424	-0.30	0.13	0.58	0.07	0.5371	-0.15	0.29	-0.27	-0.05	0.6503	-0.25	0.16	-0.68	-0.11	0.3930	-0.36	0.14
Maternal education	3.12	0.10	0.3030	-0.09	0.30	-1.16	-0.03	0.8022	-0.22	0.17	3.46	0.11	0.2407	-0.08	0.29	3.53	0.10	0.3680	-0.12	0.32
5-minute Apgar score	0.30	0.04	0.7408	-0.18	0.26	1.90	0.15	0.1775	-0.07	0.38	-0.18	-0.02	0.8443	-0.23	0.19	0.28	0.03	0.8140	-0.22	0.28
R^2					0.4112					0.3559					0.4805					0.2457
Adjusted R^2					0.3220					0.2338					0.4017					0.1314

CI, confidence interval; *p < 0.00625 (Bonferroni-corrected significance threshold for eight comparisons). B, unstandardized regression coefficient; β, standardized regression coefficient

Table S11. Sensitivity analysis: Results of multiple regression analysis on the developmental quotient in the corrected 18-month Kyoto Scale of Psychological Development after replacing parental visit frequency with the pre-/during-COVID-19 period (n = 92).

	Overall					Postural–Motor					Cognitive–Adaptive					Language–Social				
	B	β	p value	95%CI		B	β	p value	95%CI		B	β	p value	95%CI		B	β	p value	95%CI	
Gestational age	-0.75	-0.14	0.3748	-0.45	0.17	-1.05	-0.15	0.3759	-0.48	0.19	-0.82	-0.15	0.3640	-0.47	0.17	-0.04	-0.01	0.9663	-0.33	0.31
Sex	-2.98	-0.10	0.3555	-0.31	0.11	-6.18	-0.16	0.1758	-0.38	0.07	-2.14	-0.07	0.5330	-0.29	0.15	-3.61	-0.11	0.3187	-0.33	0.11
Invasive procedure count	-0.09	-0.38	0.0266	-0.71	-0.05	-0.10	-0.35	0.0585	-0.70	0.01	-0.09	-0.36	0.0380	-0.71	-0.02	-0.09	-0.34	0.0506	-0.69	0.00
Surgical history	-3.47	-0.11	0.4825	-0.43	0.20	-6.81	-0.17	0.3288	-0.51	0.17	-3.06	-0.10	0.5600	-0.42	0.23	1.39	0.04	0.8018	-0.29	0.37
Fentanyl exposure	-0.25	0.00	0.9773	-0.26	0.26	-5.82	-0.07	0.6433	-0.35	0.22	3.92	0.06	0.6790	-0.21	0.32	-6.67	-0.09	0.5050	-0.36	0.18
Midazolam exposure	0.14	0.17	0.3085	-0.16	0.49	0.03	0.03	0.8702	-0.32	0.38	0.12	0.14	0.4190	-0.20	0.47	0.19	0.21	0.2248	-0.13	0.54
Pre-/during-COVID-19 period	1.83	0.06	0.6183	-0.18	0.29	-0.64	-0.02	0.9020	-0.27	0.24	3.21	0.10	0.4120	-0.14	0.34	-1.31	-0.04	0.7499	-0.28	0.20
Age during the examination	0.11	0.01	0.9229	-0.22	0.24	3.39	0.26	0.0401	0.01	0.51	0.19	0.02	0.8780	-0.22	0.26	-0.59	-0.06	0.6476	-0.29	0.18
Maternal education	-2.71	-0.09	0.4229	-0.30	0.13	1.69	0.04	0.7226	-0.19	0.27	-4.20	-0.13	0.2430	-0.35	0.09	-0.53	-0.02	0.8885	-0.24	0.20
R^2					0.2421					0.2631					0.2183					0.1844
Adjusted R^2					0.1356					0.1595					0.1083					0.0697

CI, confidence interval; B, unstandardized regression coefficient; β, standardized regression coefficient. Bonferroni-corrected significance threshold: $p < 0.00625$.

Table S12. Sensitivity analysis: Results of multiple regression analysis on the developmental quotient in the 36-month Kyoto Scale of Psychological Development after replacing parental visit frequency with the pre-/during-COVID-19 period (n = 82).

	Overall					Postural–Motor					Cognitive–Adaptive					Language–Social				
	B	β	p value	95%CI		B	β	p value	95%CI		B	β	p value	95%CI		B	β	p value	95%CI	
Gestational age	0.09	0.02	0.9097	-0.27	0.30	-0.91	-0.11	0.4477	-0.41	0.18	-0.21	-0.04	0.7845	-0.31	0.24	0.57	0.09	0.5588	-0.22	0.41
Sex	-9.74	-0.33	0.0010*	-0.52	-0.14	-8.14	-0.18	0.0762	-0.38	0.02	-13.02	-0.42	<0.0001*	-0.61	-0.24	-6.51	-0.19	0.0777	-0.41	0.02
Invasive procedure count	-0.08	-0.38	0.0226	-0.70	-0.05	-0.10	-0.30	0.0844	-0.64	0.04	-0.10	-0.46	0.0044*	-0.77	-0.15	-0.05	-0.22	0.2337	-0.58	0.14
Surgical history	-3.94	-0.13	0.3879	-0.42	0.17	-13.48	-0.29	0.0653	-0.61	0.02	-4.77	-0.15	0.2945	-0.44	0.13	-1.90	-0.05	0.7448	-0.39	0.28
Fentanyl exposure	4.06	0.08	0.4707	-0.14	0.30	-2.48	-0.03	0.7801	-0.26	0.20	8.64	0.16	0.1266	-0.05	0.38	2.03	0.04	0.7779	-0.21	0.28
Midazolam exposure	-0.01	-0.01	0.9454	-0.22	0.20	0.07	0.04	0.7290	-0.18	0.26	-0.04	-0.03	0.7427	-0.24	0.17	0.01	0.01	0.9319	-0.23	0.25
Pre-/during-COVID-19 period	5.36	0.18	0.1150	-0.04	0.40	-0.07	0.00	0.9899	-0.24	0.23	2.34	0.08	0.4867	-0.14	0.29	8.66	0.25	0.0481	0.00	0.50
Age during the examination	-0.60	-0.11	0.3066	-0.32	0.10	0.77	0.09	0.4073	-0.13	0.31	-0.26	-0.05	0.6580	-0.25	0.16	-0.99	-0.16	0.1877	-0.39	0.08
Maternal education	2.64	0.09	0.3562	-0.10	0.27	-0.89	-0.02	0.8435	-0.21	0.18	2.67	0.08	0.3498	-0.09	0.26	3.18	0.09	0.3863	-0.12	0.30
R^2					0.4315					0.3189					0.4731					0.2910
Adjusted R^2					0.3573					0.2287					0.4044					0.1985

CI, confidence interval; *p < 0.00625 (Bonferroni-corrected significance threshold for eight comparisons). B, unstandardized regression coefficient; β, standardized regression coefficient

Table S13. Sensitivity analysis: Results of multiple regression analysis on the developmental quotient in the corrected 18-month Kyoto Scale of Psychological Development after replacing parental visit frequency with kangaroo care frequency (n = 92).

	Overall					Postural–Motor					Cognitive–Adaptive					Language–Social				
	B	β	p value	95%CI		B	β	p value	95%CI		B	β	p value	95%CI		B	β	p value	95%CI	
Gestational age	-0.81	-0.15	0.3543	-0.47	0.17	-1.18	-0.17	0.3392	-0.51	0.18	-0.91	-0.16	0.3307	-0.50	0.17	0.16	0.03	0.8672	-0.30	0.36
Sex	-2.81	-0.09	0.3792	-0.30	0.12	-6.43	-0.16	0.1550	-0.39	0.06	-1.82	-0.06	0.5929	-0.27	0.16	-3.53	-0.11	0.3227	-0.32	0.11
Invasive procedure count	-0.09	-0.39	0.0206	-0.72	-0.06	-0.10	-0.34	0.0576	-0.70	0.01	-0.09	-0.39	0.0268	-0.72	-0.05	-0.08	-0.33	0.0529	-0.67	0.00
Surgical history	-4.39	-0.14	0.3677	-0.45	0.17	-7.06	-0.17	0.3036	-0.51	0.16	-4.62	-0.14	0.3740	-0.47	0.18	2.67	0.08	0.6220	-0.24	0.40
Fentanyl exposure	0.09	0.00	0.9916	-0.26	0.26	-6.09	-0.07	0.6264	-0.35	0.21	4.55	0.06	0.6315	-0.20	0.33	-6.76	-0.09	0.4955	-0.36	0.18
Midazolam exposure	0.15	0.18	0.2822	-0.15	0.50	0.03	0.03	0.8774	-0.32	0.38	0.13	0.15	0.3705	-0.18	0.49	0.19	0.20	0.2349	-0.13	0.53
Kangaroo care frequency	-36.45	-0.04	0.7367	-0.27	0.19	-56.05	-0.05	0.7130	-0.29	0.20	-56.76	-0.06	0.6232	-0.30	0.18	101.35	0.10	0.4025	-0.14	0.34
Age during the examination	0.14	0.01	0.9049	-0.22	0.25	3.38	0.26	0.0402	0.01	0.51	0.23	0.02	0.8487	-0.22	0.26	-0.61	-0.06	0.6345	-0.29	0.18
Maternal education	-2.34	-0.07	0.4899	-0.29	0.14	1.85	0.04	0.6972	-0.18	0.27	-3.58	-0.11	0.3214	-0.33	0.11	-1.12	-0.03	0.7670	-0.25	0.19
R^2					0.2421					0.2645					0.2129					0.1920
Adjusted R^2					0.1356					0.1611					0.1023					0.0784

CI, confidence interval; B, unstandardized regression coefficient; β, standardized regression coefficient. Bonferroni-corrected significance threshold: $p < 0.00625$.

Table S14. Sensitivity analysis: Results of multiple regression analysis on the developmental quotient in the 36-month Kyoto Scale of Psychological Development after replacing parental visit frequency with kangaroo care frequency (n = 82).

	Overall					Postural–Motor					Cognitive–Adaptive					Language–Social				
	B	β	p value	95%CI		B	β	p value	95%CI		B	β	p value	95%CI		B	β	p value	95%CI	
Gestational age	-0.36	-0.07	0.6413	-0.35	0.22	-1.36	-0.17	0.2723	-0.47	0.14	-0.56	-0.10	0.4696	-0.38	0.18	0.14	0.02	0.8885	-0.31	0.35
Sex	-10.32	-0.35	0.0004*	-0.53	-0.16	-9.13	-0.20	0.0453	-0.40	0.00	-13.65	-0.44	<0.0001*	-0.63	-0.26	-6.75	-0.20	0.0700	-0.41	0.02
Invasive procedure count	-0.09	-0.43	0.0074	-0.75	-0.12	-0.10	-0.31	0.0679	-0.65	0.02	-0.11	-0.49	0.0019*	-0.80	-0.19	-0.07	-0.29	0.1188	-0.65	0.08
Surgical history	-7.38	-0.24	0.0928	-0.52	0.04	-15.02	-0.33	0.0347	-0.63	-0.02	-6.74	-0.21	0.1245	-0.49	0.06	-6.61	-0.19	0.2489	-0.52	0.14
Fentanyl exposure	3.36	0.07	0.5431	-0.15	0.28	-3.17	-0.04	0.7175	-0.27	0.19	8.07	0.15	0.1469	-0.06	0.36	1.39	0.02	0.8482	-0.22	0.27
Midazolam exposure	0.03	0.02	0.8110	-0.18	0.23	0.08	0.04	0.6907	-0.17	0.26	-0.02	-0.02	0.8583	-0.21	0.18	0.07	0.05	0.6646	-0.18	0.28
Kangaroo care frequency	-453.85	-0.22	0.0199	-0.41	-0.04	-398.74	-0.13	0.1967	-0.33	0.07	-332.33	-0.16	0.0855	-0.34	0.02	-484.31	-0.21	0.0567	-0.42	0.01
Age during the examination	-0.54	-0.10	0.3419	-0.30	0.10	0.65	0.08	0.4652	-0.14	0.29	-0.27	-0.05	0.6298	-0.24	0.15	-0.81	-0.13	0.2719	-0.36	0.10
Maternal education	3.29	0.11	0.2405	-0.07	0.29	-0.76	-0.02	0.8653	-0.21	0.18	3.04	0.10	0.2787	-0.08	0.27	4.08	0.12	0.2678	-0.09	0.32
R^2					0.4553					0.3355					0.4918					0.2882
Adjusted R^2					0.3843					0.2476					0.4255					0.1953

CI, confidence interval; *p < 0.00625 (Bonferroni-corrected significance threshold for eight comparisons). B, unstandardized regression coefficient; β, standardized regression coefficient

Table S15. Sensitivity analysis: Results of multiple regression analysis on the developmental quotient in the 36-month Kyoto Scale of Psychological Development after additional adjustment for the developmental quotient at corrected 18 months (n = 82).

	Overall					Postural–Motor					Cognitive–Adaptive					Language–Social				
	B	β	p value	95%CI		B	β	p value	95%CI		B	β	p value	95%CI		B	β	p value	95%CI	
Gestational age	0.64	0.12	0.2909	-0.10	0.34	-0.40	-0.05	0.7330	-0.34	0.24	0.36	0.07	0.5553	-0.16	0.29	1.21	0.20	0.1749	-0.09	0.49
Sex	-6.78	-0.23	0.0042*	-0.38	-0.07	-5.98	-0.13	0.1810	-0.33	0.06	-10.91	-0.36	<0.0001*	-0.51	-0.20	-2.43	-0.07	0.4717	-0.27	0.13
Invasive procedure count	-0.03	-0.12	0.3519	-0.38	0.14	-0.03	-0.09	0.5960	-0.42	0.25	-0.05	-0.21	0.1092	-0.47	0.05	0.00	0.00	0.9837	-0.33	0.34
Surgical history	-3.22	-0.11	0.3600	-0.33	0.12	-10.90	-0.24	0.1100	-0.53	0.06	-2.22	-0.07	0.5372	-0.30	0.16	-2.72	-0.08	0.5998	-0.37	0.22
Fentanyl exposure	0.03	0.00	0.9957	-0.25	0.25	-5.92	-0.08	0.6280	-0.40	0.24	3.99	0.08	0.5404	-0.17	0.32	-1.43	-0.02	0.8791	-0.35	0.30
Midazolam exposure	-0.05	-0.04	0.6453	-0.21	0.13	0.00	0.00	0.9820	-0.22	0.22	-0.10	-0.08	0.3247	-0.25	0.09	-0.01	-0.01	0.9348	-0.23	0.21
Parental visit frequency	0.00	0.00	0.9943	-0.16	0.17	0.03	0.03	0.7500	-0.18	0.25	-0.05	-0.08	0.3202	-0.25	0.08	0.04	0.05	0.6142	-0.16	0.27
Age during the examination	-0.08	-0.01	0.8673	-0.18	0.15	1.13	0.14	0.1990	-0.07	0.34	0.04	0.01	0.9338	-0.16	0.17	-0.31	-0.05	0.6471	-0.26	0.16
Maternal education	5.19	0.17	0.0298	0.02	0.32	-0.25	-0.01	0.9560	-0.20	0.19	5.01	0.16	0.0402	0.01	0.31	6.04	0.17	0.0843	-0.02	0.37
Developmental quotient at corrected 18 months	0.66	0.61	<0.0001*	0.45	0.78	0.74	0.46	<0.0001*	0.25	0.67	0.59	0.53	<0.0001*	0.37	0.70	0.74	0.61	<0.0001*	0.39	0.82
R^2					0.6959					0.4757					0.6989					0.5120
Adjusted R^2					0.6452					0.3868					0.6487					0.4306

CI, confidence interval; *p < 0.00625 (Bonferroni-corrected significance threshold for eight comparisons). B, unstandardized regression coefficient; β, standardized regression coefficient

Table S16. Sensitivity analysis: Results of multiple regression analysis on the developmental quotient in the corrected 18-month Kyoto Scale of Psychological Development after excluding influential cases identified by Cook's distance (n = 79).

	Overall					Postural–Motor					Cognitive–Adaptive					Language–Social				
	B	β	p value	95%CI		B	β	p value	95%CI		B	β	p value	95%CI		B	β	p value	95%CI	
Gestational age	-0.73	-0.14	0.3864	-0.45	0.18	-1.07	-0.15	0.3702	-0.49	0.18	-0.79	-0.14	0.3839	-0.46	0.18	-0.05	-0.01	0.9570	-0.33	0.31
Sex	-2.87	-0.09	0.3764	-0.31	0.12	-5.91	-0.15	0.1958	-0.38	0.08	-1.92	-0.06	0.5780	-0.28	0.16	-3.79	-0.12	0.2971	-0.33	0.10
Invasive procedure count	-0.09	-0.38	0.0246	-0.72	-0.05	-0.11	-0.35	0.0525	-0.71	0.00	-0.09	-0.37	0.0336	-0.72	-0.03	-0.08	-0.33	0.0564	-0.68	0.01
Surgical history	-3.76	-0.12	0.4485	-0.44	0.20	-7.36	-0.18	0.2917	-0.52	0.16	-3.60	-0.11	0.4954	-0.44	0.21	1.81	0.05	0.7440	-0.27	0.38
Fentanyl exposure	-0.20	0.00	0.9821	-0.27	0.26	-5.10	-0.06	0.6869	-0.34	0.23	4.05	0.06	0.6731	-0.21	0.33	-6.95	-0.09	0.4914	-0.37	0.18
Midazolam exposure	0.15	0.17	0.2913	-0.15	0.50	0.03	0.03	0.8592	-0.32	0.38	0.13	0.15	0.3864	-0.19	0.48	0.19	0.20	0.2357	-0.13	0.54
Parental visit frequency	-0.02	-0.03	0.7973	-0.27	0.21	0.04	0.06	0.6707	-0.20	0.32	-0.03	-0.05	0.6914	-0.30	0.20	0.00	0.00	0.9879	-0.25	0.25
Age during the examination	0.15	0.01	0.9000	-0.22	0.25	3.36	0.26	0.0413	0.01	0.51	0.25	0.02	0.8414	-0.22	0.26	-0.61	-0.06	0.6373	-0.30	0.18
Maternal education	-2.36	-0.07	0.4871	-0.29	0.14	1.30	0.03	0.7844	-0.20	0.26	-3.60	-0.11	0.3199	-0.33	0.11	-0.69	-0.02	0.8549	-0.24	0.20
R^2					0.2400					0.2650					0.2119					0.1831
Adjusted R^2					0.1331					0.1617					0.1011					0.0682

CI, confidence interval; B, unstandardized regression coefficient; β, standardized regression coefficient. Bonferroni-corrected significance threshold: $p < 0.00625$.

Table S17. Sensitivity analysis: Results of multiple regression analysis on the developmental quotient in the 36-month Kyoto Scale of Psychological Development after excluding influential cases identified by Cook's distance (n = 67).

	Overall					Postural–Motor					Cognitive–Adaptive					Language–Social				
	B	β	p value	95%CI		B	β	p value	95%CI		B	β	p value	95%CI		B	β	p value	95%CI	
Gestational age	0.84	0.16	0.2777	-0.13	0.45	0.05	0.01	0.9677	-0.29	0.30	0.66	0.12	0.4163	-0.17	0.41	1.18	0.19	0.2166	-0.12	0.50
Sex	—	—	—	—	—	—	—	—	—	—	—	—	—	—	—	—	—	—	—	—
Invasive procedure count	-0.10	-0.48	0.0033*	-0.79	-0.17	-0.14	-0.44	0.0074	-0.75	-0.12	-0.11	-0.51	0.0019*	-0.83	-0.20	-0.08	-0.33	0.0527	-0.67	0.00
Surgical history	—	—	—	—	—	—	—	—	—	—	—	—	—	—	—	—	—	—	—	—
Fentanyl exposure	7.45	0.10	0.2158	-0.09	0.38	0.31	0.00	0.9726	-0.23	0.24	13.14	0.25	0.0382	0.01	0.49	4.33	0.07	0.5559	-0.18	0.33
Midazolam exposure	-0.03	0.00	0.8278	-0.24	0.19	-0.03	-0.02	0.8639	-0.24	0.20	-0.08	-0.07	0.5545	-0.28	0.15	0.02	0.02	0.8891	-0.22	0.25
Parental visit frequency	-0.01	-0.04	0.8920	-0.23	0.20	-0.02	-0.02	0.8523	-0.24	0.20	-0.03	-0.06	0.6035	-0.28	0.16	0.01	0.02	0.8592	-0.21	0.25
Age during the examination	-0.59	-0.04	0.3425	-0.33	0.12	0.52	0.06	0.5820	-0.16	0.29	-0.47	-0.08	0.4725	-0.31	0.14	-0.76	-0.12	0.3213	-0.36	0.12
Maternal education	1.49	0.13	0.6282	-0.15	0.25	-2.59	-0.06	0.5764	-0.26	0.14	0.97	0.03	0.7613	-0.17	0.23	2.67	0.08	0.4797	-0.14	0.29
R^2					0.3148					0.2517					0.3068					0.2205
Adjusted R^2					0.2472					0.1769					0.2384					0.1437

CI, confidence interval; *p < 0.00625 (Bonferroni-corrected significance threshold for eight comparisons). B, unstandardized regression coefficient; β, standardized regression coefficient. — Not estimable because only one category was represented in the sensitivity analysis dataset.
